# Circulatory Metabolomics Reveals the Association of the Metabolites With Clinical Features in the Patients With Intrahepatic Cholestasis of Pregnancy

**DOI:** 10.3389/fphys.2022.848508

**Published:** 2022-07-11

**Authors:** Wenhu Liu, Qiang Wang, Jinxia Chang, Anup Bhetuwal, Nisha Bhattarai, Xin Ni

**Affiliations:** ^1^ Department of Gynecology and Obstetrics, International Collaborative Research Center for Medical Metabolomics, National Clinical Research Center for Geriatric Disorders, Xiangya Hospital Central South University, Changsha, China; ^2^ School of Pharmacy, School of Basic Medical Sciences and Forensic Medical, North Sichuan Medical College, Nanchong, China; ^3^ Department of Laboratory Medicine, Translational Medicine Research Center, North Sichuan Medical College, Nanchong, China; ^4^ Department of Clinical Laboratory, Affiliated Hospital of North Sichuan Medical College, Nanchong, China; ^5^ Department of Radiology, Affiliated Hospital of North Sichuan Medical College, Nanchong, China; ^6^ Department of Neurology, Affiliated Hospital of North Sichuan Medical College, Nanchong, China

**Keywords:** intrahepatic cholestasis of pregnancy, circulatory metabolomics, clinical features, pregnancy outcomes, bile acid

## Abstract

**Objective:** Intrahepatic cholestasis of pregnancy (ICP) is associated with an increased risk of adverse pregnancy to the mother and fetus. As yet, the metabolic profiles and the association of the clinical features remain obscure.

**Methods:** Fifty-seven healthy pregnant women and 52 patients with ICP were recruited in this study. Plasma samples were collected from pregnancies who received prenatal care between 30 and 36 weeks. Untargeted metabolomics to portray the metabolic profiles were performed by LC/MS. Multivariate combined with the univariate analysis was performed to screen out differential metabolites between the ICP and control groups. A de-biased sparse partial correlation (DSPC) network analysis of differential metabolites was conducted to explore the potential mutual regulation among metabolites on the basis of de-sparsified graphical lasso modeling. The pathway analysis was carried out using MetaboAnalyst. Linear regression and Pearson correlation analysis was applied to analyze correlations of bile acid levels, metabolites, newborn weights, and pregnancy outcomes in ICP patients.

**Results:** Conspicuous metabolic changes and choreographed metabolic profiles were disclosed: 125 annotated metabolites and 18 metabolic pathways were disturbed in ICP patients. DSPC networks indicated dense interactions among amino acids and their derivatives, bile acids, carbohydrates, and organic acids. The levels of total bile acid (TBA) were increased in ICP patients with meconium-stained amniotic fluid (MSAF) compared with those without MSAF. An abnormal tryptophan metabolism, elevated long chain saturated fatty acids and estrone sulfate levels, and a low-antioxidant capacity were relevant to increased bile acid levels. Newborn weights were significantly associated with the levels of bile acids and some metabolites of amino acids.

**Conclusion:** Our study revealed the metabolomic profiles in circulation and the correlation of the metabolites with clinical features in ICP patients. Our data suggest that disturbances in metabolic pathways might be associated with adverse pregnancy outcomes.

## Introduction

Intrahepatic cholestasis of pregnancy (ICP), a pregnancy-specific liver disease with its onset typically in the second or third trimester, is characterized by maternal pruritus, jaundice, and disorders of metabolism accompanied by elevated serum bile acids and liver transaminases (Ovadia et al., 2019). The incidence of ICP varies from 0.1 to 15.6% with ethnicity and regional disparity (Ozkan et al., 2015). Although ICP can revert spontaneously after labor, the development of ICP has been implicated to be closely linked with a higher risk of adverse pregnancy outcomes to the fetus, including premature labor, meconium-stained amniotic fluid (MSAF), and respiratory distress. These adverse effects particularly occur in ICP cases with serum bile acid levels that exceed 40 μmol/L (Kawakita et al., 2015; Ovadia et al., 2019).

Although the definite etiology of ICP is still obscure, it has been implicated that genetic predisposition, hormonal, and environmental factors contribute to the development of ICP. For instance, the genetic mutation of bile salt export pump (*ABCB11*) and an abnormal expression of bile acid farnesoid X receptor (FXR) along with its target genes contribute to the occurrence of ICP by affecting lipid and glucose metabolic homeostasis, which might interfere with the normal metabolism of bile acids (Dixon et al., 2009; Song et al., 2014). Estrogen and progesterone metabolites might contribute to the development of ICP by inducing a trans-inhibition of the bile salt export pump and the subsequent toxicity due to the accumulation of bile acids (Vallejo et al., 2006). As for environmental factors, low dietary selenium could be a risk factor because it reduces the activity of selenoenzyme glutathione peroxidase and subsequently decreases the anti-oxidant capacity of the liver (Reyes et al., 2000). Some studies have demonstrated that ICP is associated with an impaired glycolipid metabolism (Martineau et al., 2015).

Given that ICP is a pregnancy-associated liver disease, it would be of great interest to investigate the metabolic state *in vivo*. Metabolomics is generally used to quantitatively analyze the changes of metabolites in biofluids of the patients with various diseases in order to understand their metabolic state *in vivo*. A recent study on urinary metabolomics of ICP patients shows a marker panel composed of five metabolites including 1-monoacylglyceride (22:5), lysophosphatidylethanolamine (22:5), homocysteine sulfonic acid, glycocholic acid, and chenodeoxycholic acid 3-sulfate (Ma et al., 2017). Using targeted metabolomics, Li et al. (2018) have demonstrated that the urinary levels of sulfated bile acids (SBAs) are remarkably increased in ICP patients and the levels of glycine cholic acid 3-sulfate (GCA-3S) and sulfated dihydroxy glycine bile acid (di-GBA-S) are correlated with ICP severity, suggesting that these SBAs could be a potential biomarker for the diagnosis and grading of ICP. More recently, Dong *et al.* (Dong et al., 2021) have shown placental metabolomics in ICP patients. However, there are few studies regarding circulatory metabolomic profiles in ICP patients.

In the present study, untargeted metabolomics was implemented to profile metabolites in circulation of ICP patients between 30 and 36 gestational weeks. It was found that 125 annotated metabolites are altered prominently and 18 metabolic pathways are disturbed clearly in ICP patients. Bile acid levels are increased in ICP patients with meconium contamination in the amniotic fluid. Moreover, abnormal tryptophan metabolism, elevated estrone sulfate, and long chain saturated fatty acid (LCSFA) levels and a low-antioxidant capacity are pertinent to increased bile acid levels. Bile acid levels are also associated with newborn weight, estrone sulfate, along with a number of amino acids and their metabolites. Our data provide new information regarding the metabolic state and potential biomarkers for adverse pregnancy outcomes in ICP patients.

## Materials and Methods

### Ethical Considerations

This study was approved by the Ethics Committee of the North Sichuan Medical College (No: 202,103). All procedures performed in this study regarding the participants were in accordance with the ethical standards of the institution and with the Declaration of Helsinki (World Medical Association, 2013). Informed consent was signed by all participants.

### Study Cohort and Sampling

Fifty-seven healthy pregnant women (control group) and 52 patients with ICP (ICP group) were recruited from the affiliated hospital of North Sichuan Medical College (Nanchong, Sichuan, China) from July 2019 to June 2020. Maternal blood samples were collected from pregnancies who received prenatal care between 30 and 36 weeks. ICP was diagnosed according to the guidelines for ICP diagnosis, i.e., circulatory total bile acid (TBA) level >10 μmol/L (Brouwers et al., 2015). The patients who had a history of liver diseases, cholelithiasis, diabetes, hypertension, or other skin diseases were not included in the present study. Infants with congenital malformations, deformations, or chromosomal abnormalities were excluded. In the ICP group, 9 patients received ursodeoxycholic acid (UDCA) treatment. Gestational age-matched pregnant women without co-morbidities were enrolled during the same period as the control group. Pregnancy outcomes including stillbirth, gestational age at delivery, premature rupture of membrane (PROM), meconium-stained amniotic fluid (MSAF), newborn’s Apgar score, newborn’s weight, and postpartum hemorrhage were recorded in both groups. Maternal demographic data and blood biochemical data were collected at the same time.

The fasting blood sample from each individual was collected into a vacutainer EDTA anticoagulated tube. The samples were then centrifuged (1500 g) for 10 min at 4°C. The plasma was collected and then stored at −80°C for the following analysis. 100 μL of each sample was mixed with 400 μL pre-cold solvent (methanol/acetonitrile = 1:1, *V/V*), and then vortexed for 1 min and incubated for 1 h at −20°C. The supernatants were collected by centrifugation (14,000 g) for 15 min at 4°C, and lyophilized by using a vacuum drying concentrator and stored at -80°C. The extracts were then re-suspended in 100 μL of a solvent containing water/acetonitrile (1:1, *V/V*) and vortexed for 30 s and centrifuged (14,000 g) for 15 min at 4°C. Supernatants were collected and transferred to autosampler vials for the LC/MS analysis. QC samples were obtained by mixing equal volumes (20 μL) from all the samples.

### UPLC-Q-TOF/MS Analysis, Data Analysis, and Metabolite Identification

Metabolomic profiling was conducted by Shanghai Applied Protein Technology, Ltd. (Shanghai, China) *via* liquid chromatography tandem mass spectrometry (LC-MS/MS). Analyses were performed using an UHPLC (1290 Infinity LC, Agilent Technologies) coupled to a quadrupole time-of-flight (AB Sciex TripleTOF 6600). Chromatographic separation was conducted with a HILIC column (2.1 mm × 100 mm, 1.7 µm, ACQUITY UPLC BEH Amide, Waters). The temperature of the UPLC column oven was maintained at 25°C while the autosampler was set at 4°C. The mobile phase contained: (A) water with 25 mM ammonia and 25 mM ammonium acetate, and (B) acetonitrile. The elution gradient was set as follows: initial elution with 95% B for 0.5 min; linearly changed B from 95 to 65% for 0.5–7 min; linearly changed B from 65 to 40% for 7–8 min; 40% B maintained for 8–9 min; linearly decreased B from 40 to 95% for 9–9.1 min; and 95% B maintained for 9.1–12 min. The flow rate was 300 μL/min. The injection volume of each sample was 2 μL. Solvent blanks and QC samples were inserted into the analytical batch every ten samples to assess the stability of the instrument’s performance. The stability of the system was assessed *via* the coefficient of variation (CV) of each variable in the QC sample.

The Q-TOF/MS 6600 mass spectrometer was applied to carry out mass spectrometry equipped with dual electrospray ionization (ESI). ESI source parameters were set as follows: Curtain gas at 30 psi, gas1 and gas2 of an ion source at 60 psi, ion spray voltage floating at +5500V for ESI+, and –5500 V for the ESI- mode, respectively. Source temperature was set at 600°C. The operating parameters were set as follows: MS scan range of *m/z* 60–1,000, and production scan range of *m/z* 25–1,000. The full scan accumulation time was set at 0.20 s/spectra, and product ion scan at 0.05 s/spectra. Data acquisition was performed in the mode of information-dependent acquisition (IDA). Candidate ions to monitor per cycle were 6, and the excluded isotope was within 4Da. Collision energy was 35 ± 15 eV. Declustering potential was +60V for the ESI + mode, and –60V for the ESI–mode, respectively.

The raw MS data (wiff scan files) were converted to mzXML files using ProteoWizard MS Convert before importing into the freely available XCMS software. Compound identification of the metabolites by using the MS/MS spectra with an in-house database was established with available authentic standards. The identifications were manually checked to exclude exogenous compounds.

The peak area of each variable was normalized by dividing the total area of the sample after which values were multiplied by 10^5^ for the ease of presentation. Variables with CV over 30% were deleted in both modes. For the multivariate data analysis (MVDA), center and Pareto scaling were implemented to all data ahead of MVDA to reduce the influence of maximal and minimal values and the noise effects in models. A principal component analysis (PCA) was performed to show the clustering trend and classified information of the samples. Subsequently, the partial least squares-discriminant analysis (PLS-DA) was used to distinguish a UDCA-treated group from an untreated group based on metabolic characteristics. An orthogonal partial least square discriminant analysis (OPLS-DA) was applied to maximize the differences of metabolic profiles between the control and ICP groups. The variable importance in the projection (VIP) scores was calculated according to the OPLS-DA models. The 7-fold cross-validation and permutation testing was applied to evaluate the predictive ability of the models, respectively. Differential metabolites were selected by the multivariate combined with the univariate analysis according to the following criteria: VIP >1.0, FC ≥ 1.2 or ≤0.83, *p* < 0.05.

### Analysis of the De-Biased Sparse Partial Correlation Network of Differential Metabolites

A de-biased sparse partial correlation (DSPC) weighted network analysis of differential metabolites was performed on the basis of de-sparsified graphical lasso modeling procedure (Basu et al., 2017) and visualized by Cytoscape software (version 3.7.1). The nodes represent metabolites while the edges represent the partial correlation coefficients. Lasso modeling procedure was used to reduce the confounders’ association coefficient and thus limit spurious correlations in these networks. *p* values were adjusted and the specific significant cutoff was set to 0.05 according to the Benjamini–Hochberg method. The specific range for correlation coefficients was from −1 to 1. The default DSPC network only showed the top correlation (edges) based on their *p* value ranking (top 20% when the total number of edges is less than or the top 100 edges when the total number of edges is greater than 1,000).

### Statistical Analysis

The statistical analysis was accomplished using the IBM SPSS 23.0 package (IBM SPSS, Turkey). Normality of the data distributions was tested using the Shapiro–Wilks test. The data with normal distribution were analyzed by using the unpaired Student’s *t-*test, while non-normally distributed data were analyzed by the non-parametric Mann–Whitney *U* test. Categorical variables were analyzed through the Chi-Square test. The correlation coefficient was assessed using Pearson and distance correlation analyses. *p* < 0.05 was considered statistically significant. Data were expressed as mean ± SEM (normal distribution data) or medians and ranges (deviation from a normal distribution data), and displayed using GraphPad Prism 8.0.1 (GraphPad Prism, Inc. San Diego, United States) or R package (version 3.5.2).

## Results

### General Characteristics of Participants

Among the ICP patients recruited, there were 40 patients with TBA levels at 10–39.9 μmol/L and 12 patients with TBA levels>40 μmol/L. As shown in Table 1, TBA, MSAF, newborn’s weight, aspartate aminotransferase (AST), alanine aminotransferase (ALT), alkaline phosphatase (ALP), gamma-glutamyl transferase (GGT), albumin (ALB), total bilirubin (TBIL), and direct bilirubin (DBIL) levels were significantly altered in ICP patients compared with those of the control group (*p* < 0.05). There was no statistical difference in the levels of maternal age, body mass index (BMI), spontaneous vaginal birth, gestational age at delivery, PROM, total protein (TP), and globulin (GLB) between the two groups. Among the 12 patients with a high TBA level (>40 μmol/L), MSAF was detected in 6 patients. Among 40 ICP patients with TBA level <40 μmol/L, 4 patients (10%) had MSAF.

**TABLE 1:** Obstetric information and biochemical results of the participants in the control and ICP groups.

Baseline information	Control (*n* = 57)	ICP (*n* = 52)	*p* value
Maternal age (y)^a^	29.86 ± 3.96	30.21 ± 2.94	0.606
Stillbirth (%)	0	0	–
BMI (kg/m^2^)^a^	26.70 ± 2.34	26.29 ± 2.11	0.348
Spontaneous vaginal birth (*n*) (%)^b^	23 (40.35%)	17 (32.69)	0.529
Gestational age at delivery (wk)^a^	38.64 ± 5.59	37.86 ± 6.70	0.362
Twins (*n*) (%)^b^	4 (7.0%)	6 (11.5%)	0.514
Apgar score>8 (*n*) (%)	57 (100%)	52 (100%)	–
PROM (*n*) (%)^b^	4 (7.0%)	2 (3.8%)	0.681
MSAF (*n*) (%)^b^	4 (7.0%)	10 (19.2%)	<0.01
Newborn weight (g)^a^	3386.52 ± 207.31	3154.12 ± 156.99	<0.001
Postpartum hemorrhage (*n*) (%)	0	0	–
TBA (μmol/L)^c^	2.9 (1.8–5.2)	22.2 (15.43–34.65)	<0.001
AST (U/L)^c^	19 (17–27.5)	57 (19–187)	<0.001
ALT (U/L)^c^	15 (10–34.5)	83 (17–302)	<0.001
ALP (U/L)^c^	138 (102–176.5)	219 (134–301)	<0.001
GGT (U/L)^c^	13 (9–21)	31 (11–68)	<0.001
ALB (g/L)^c^	35.5 (33.95–37)	34 (31.6–35.9)	0.014
TBIL (μmol/L)^c^	8.1 (6.5–9.7)	9.9 (8.1–12.5)	<0.001
DBIL (μmol/L)^c^	1.4 (0.7–1.9)	2.1 (1.2–3.2)	<0.001
TP (g/L)^c^	68.3 (63.7–70.5)	66.6 (61.6–69.5)	0.161
GLB (g/L)^c^	31.9 (29.7–34.3)	31.7 (29.6–33.9)	0.926

a Values are expressed as mean ± SEM (Students *t*-test).

b Values are expressed as number (percentage) (*χ* ^2^ test).

c Values are expressed as median (interquartile range). BMI, body mass index; PROM, premature rupture of membrane; MSAF, meconium-stained amniotic fluid; TBA, total bile acid; AST, aspartate transaminase; ALT, alanine transaminase; ALP, alkaline phosphatase; GGT, gamma-glutamyltransferase; ALB, albumin; TBIL, total bilirubin; DBIL, direct bilirubin; TP, total protein; GLB, globulin.

### Circulatory Metabolomic Profiling in Heathy Pregnant Women and ICP Patients

**FIGURE 1:**
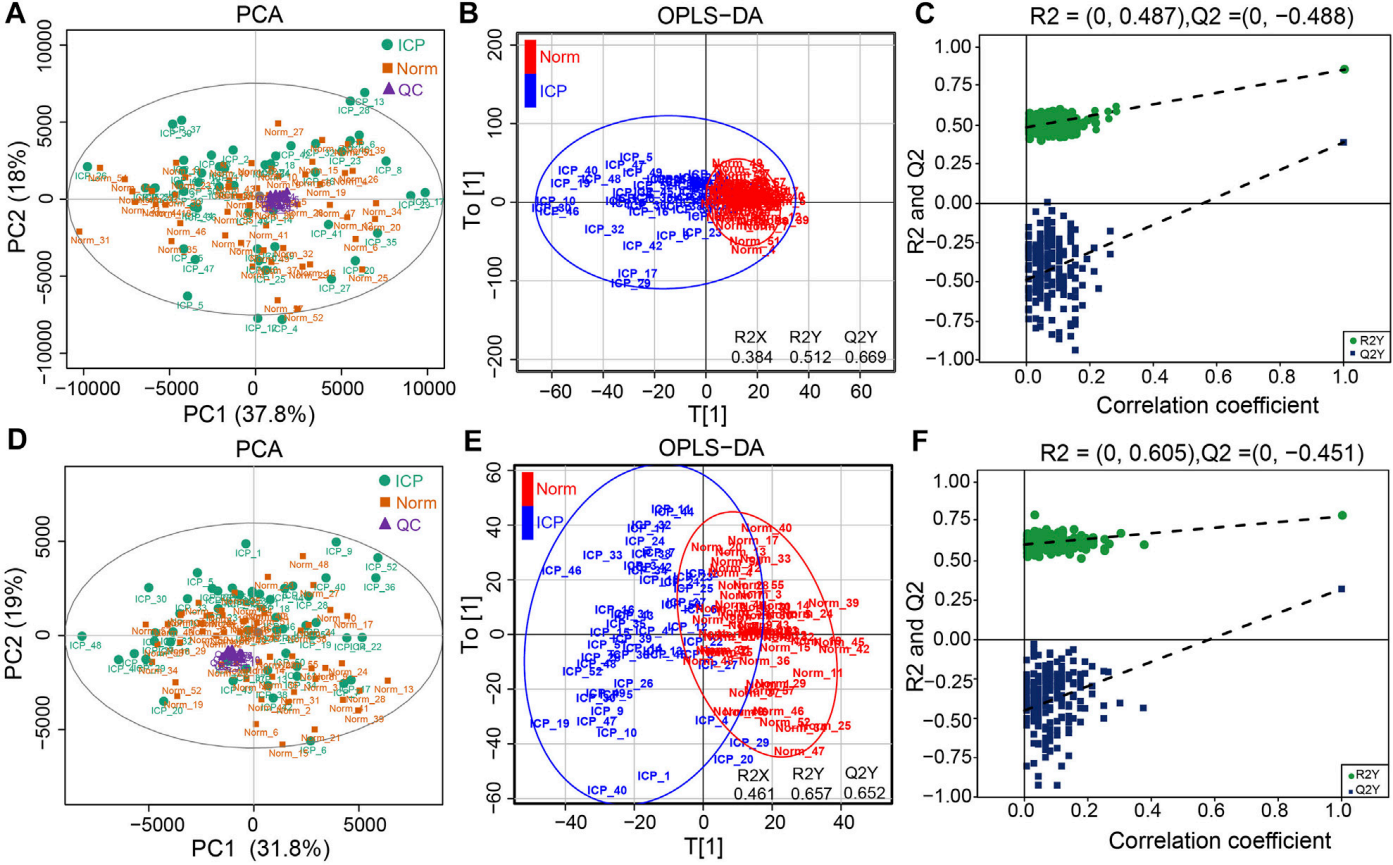
Metabolic profiles of plasma samples. **(A,D)** PCA plots of the normal and ICP groups in the ESI+ and ESI- modes, respectively. **(B,E)** OPLS-DA plots of the normal and ICP groups in both modes, respectively. **(C,F)** Permutation test plots of the normal and ICP groups in both modes, respectively.

**FIGURE 2:**
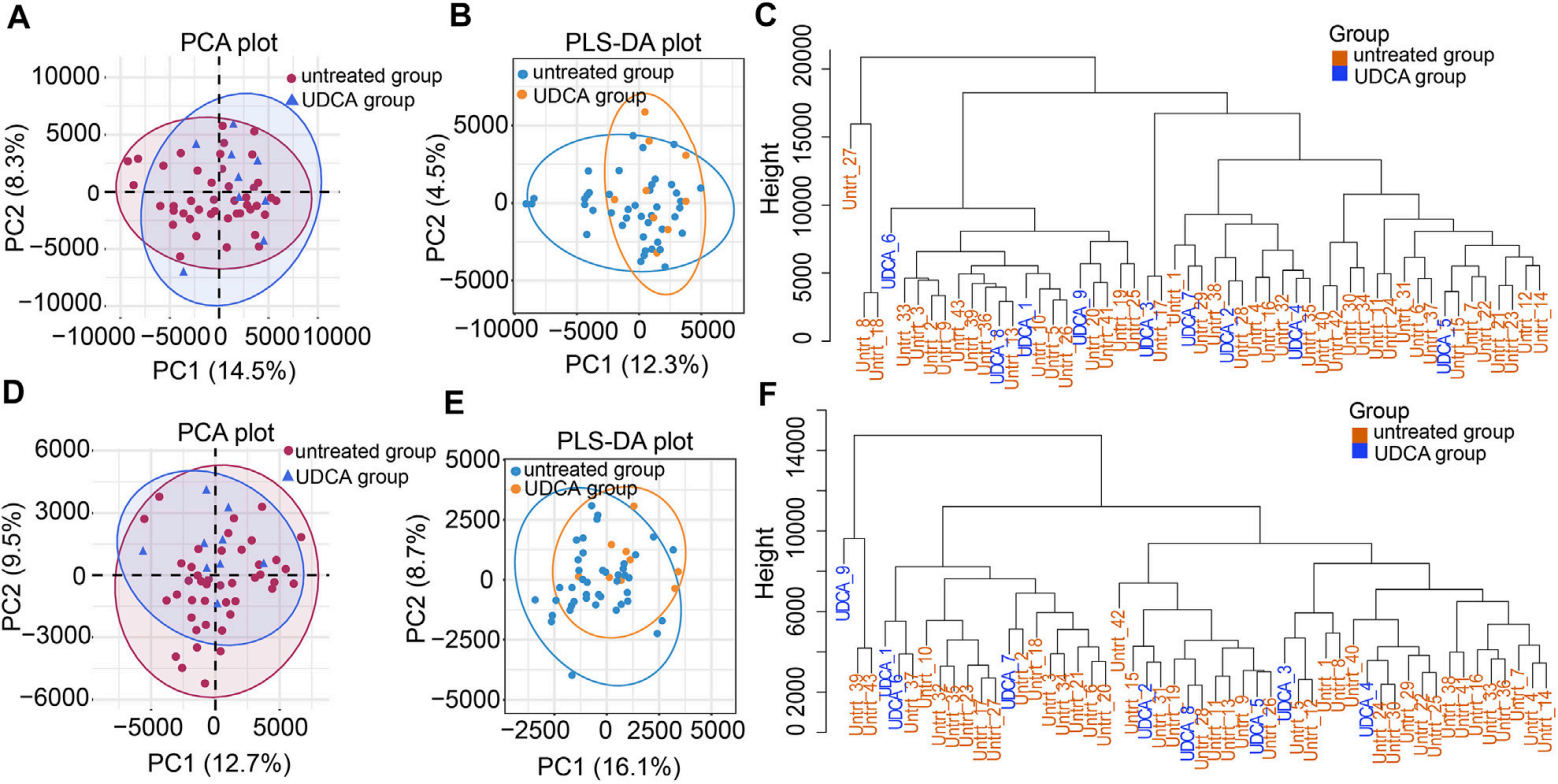
Metabolic profiles of the UDCA-treated and untreated groups. **(A,D)** PCA plots of the two groups in the ESI+ and ESI- modes, respectively. **(B,E)** PLS-DA plots of the two groups in both modes. **(C,F)** Hierarchical clustering trees of the two groups in both modes.

All the raw data of mass spectrometry have been deposited to the MetaboLights database (Accession number: MTBLS2627 accessed *via* link: https://www.ebi.ac.uk/metabolights). QC results showed good repeatability of PCA-X plots in two modes within a ±2 standard deviation (Supplementary Figures S1A,B). QC samples were clustered closely together in the principal component analysis (PCA) plot of either the ESI + or ESI- modes, respectively (Figures 1A,D). The high positive correlation (*r* ≥ 0.98) of peak areas in each QC sample displayed a good repeatability (Supplementary Figure S1C,D). We first analyzed the metabolomic profiles in ICP patients with and without UDCA treatment. Both of the unsupervised PCA and supervised PLS-DA plots show that variables were poorly separated between the two groups (Figures 2A,B,D,E). Moreover, the hierarchical cluster analysis (HCA) showed that the samples of the UDCA-treated group were randomly distributed among the samples of the untreated group in both the ESI+ and ESI- modes (Figures 2C,F). A metabolome comparison by using heatmaps indicated no significant distinction between the two groups (Supplementary Figures S2A,B). The data of all ICP patients, no matter with or without UDCA treatment, were therefore included in the group of ICP patients in the follow-up analysis.

PCA score plots showed that the data in the control and ICP patients were not well separated on the basis of the first two principal components in the two modes (Figures 1A,D). However, a clear separation along the T[1] axis could be observed in supervised OPLS-DA (Figures 1B,E), suggesting an apparently different metabolic profile between the two groups. It also showed that the classification of the control and ICP groups yielded one predictive (t1) and one orthogonal (to) component with 7-fold cross-validated predicted variance *Q*2Y of 0.669 in ESI+, and 0.652 in ESI-, respectively. In addition, the OPLS-DA modes were explained by variance *R*2X (0.384), *R*2Y (0.512) for ESI+, and *R*2X (0.461), *R*2Y (0.657) for ESI- respectively, suggesting the reliability of explanation and prediction of the classification in our models. The validity of OPLS-DA models was further evaluated with 200 random iterations, permutation tests. It showed that intercepts values were *R*2 = 0.487, *Q*2 = –0.488 for ESI+, and *R*2 = 0.605, *Q*2 = –0.451 for ESI–, respectively (Figures 1C,F), indicating that OPLS-DA models were credible and not overfitting.

### The Levels of Many Metabolites Including Bile Acids, Amino Acids, Steroid Hormones, and Lipids are Changed in ICP Patients

**FIGURE 3:**
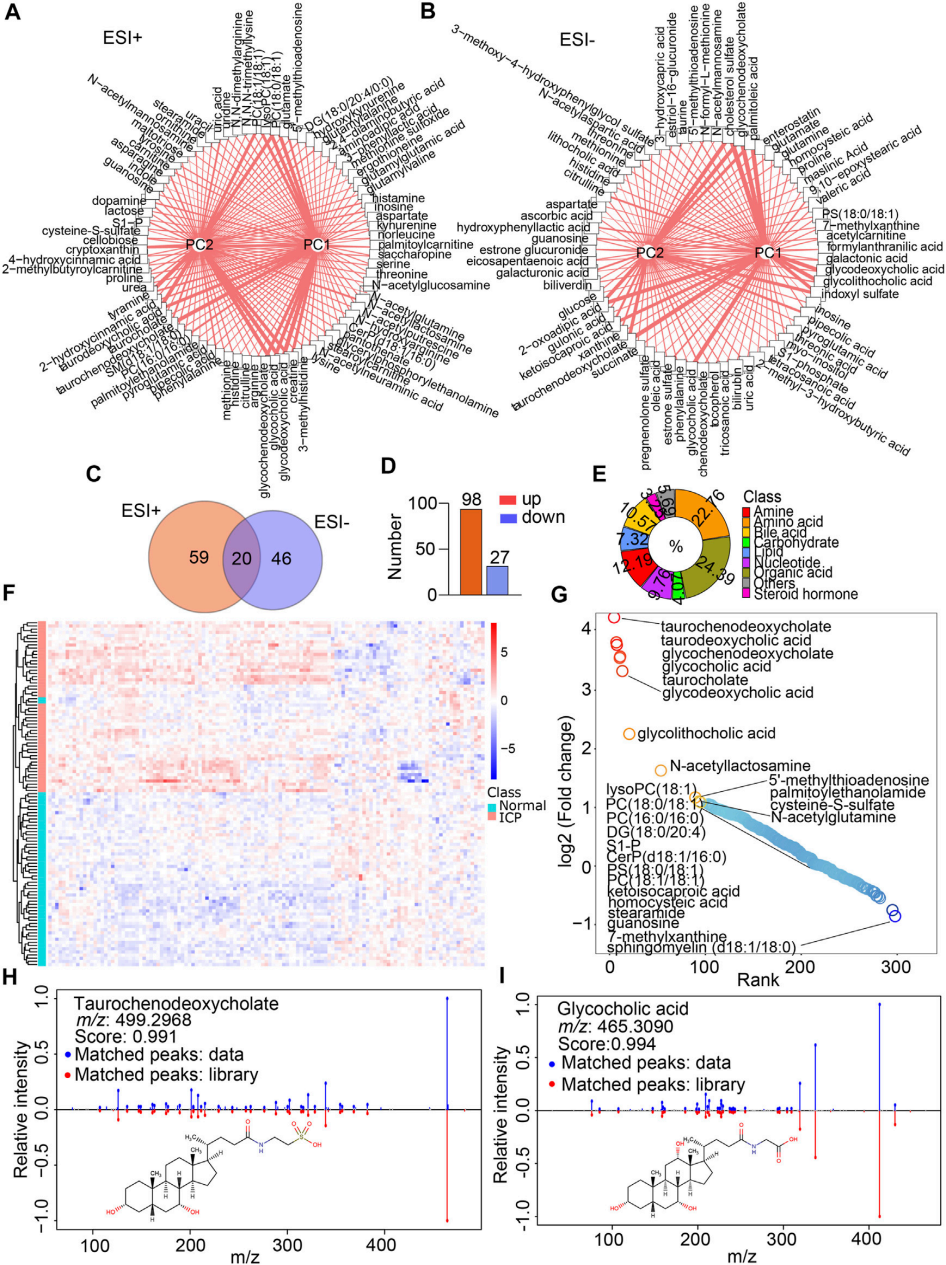
Metabolite annotation and differential metabolite screenings. **(A,B)** Network analysis of the VIP score of each differential metabolite according to the contribution for PC1 and PC2 in the ESI+ and ESI- modes, respectively. Width of lines represents the VIP scores. **(C)** Venn diagram indicates the shared and non-shared metabolites in the normal and ICP groups. **(D)** Increased and decreased metabolites in the ICP group. **(E)** Classification of metabolites by their properties. **(F)** Heatmap visualizes the differential metabolites’ signal intensities, the column represents the samples, and the row represents the metabolites. The red and blue denote the increased and decreased metabolites in the ICP group, respectively. **(G)** Rank plots show the top increased (red) and decreased (blue) metabolites in the ICP group. **(H,I)** MS/MS spectral fragments of typical metabolites (glycocholic acid and taurochenodeoxycholate) are confirmed by matching the standard library.

A total of 364 compounds was discerned and characterized. Among them, 202 and 162 ones were quantified in the ESI+ and ESI- modes, respectively. V-plots were constructed for the analysis of critical variables using the VIP values vs. coefficients of each variable to distinguish the metabolome in the two groups based on the OPLS-DA models (Supplementary Figures S3A,B). The variables with a VIP score more than 1 were considered as potential candidates, which were then visualized by score networks according to the contribution of the variables in principal component 1 (PC1) and principal component 2 (PC2) in the two modes, respectively (Figures 3A,B). Notably, the contribution of bile acids was the biggest one in PC1 and PC2 in both ESI+ and ESI- modes. Subsequently, volcano plots were plotted to recognize differential metabolites against the corresponding *p* value obtained from the Mann–Whitney *U* test (Supplementary Figures S3C,D). The Wayne chart showed that 59 annotated differential metabolites were exclusively of the ESI + mode, 46 metabolites were identified in the ESI- mode, and 20 ones were overlapped in both modes (Figure 3C). By contrast, 98 metabolites displayed more than a 1.2-fold increase whereas 27 of them showed less than a 0.83-fold decrease in the ICP group (Figure 3D, Supplementary Table S1). These metabolites included amino acids, amines, bile acids, carbohydrates, lipids, organic acids, nucleotides, steroid hormones, and others (Figure 3E), suggesting that extensive metabolites were incorporated in our data. Furthermore, the heatmap of the hierarchical cluster analysis indicated that a majority of the metabolites were increased distinctively in the ICP group (Figure 3F). Of that note, the top metabolites that increased were bile acids such as taurochenodeoxycholate, taurodeoxycholic acid, glycocholic acid, taurocholate, glycochenodeoxycholate, and glycolithocholic acid. The metabolites that decreased were lipids or lipid-like compounds, including lysophosphatidylcholine (LysoPC), phosphatidylcholines (PCs) and sphingosine 1-phosphate (S1-P), etc. (Figure 3G). The structures of metabolites with significant alterations were confirmed by matching the MS/MS spectrum in the self-built database. The representative MS/MS figures are shown in Figures 3H,I.

### De-Biased Sparse Partial Correlation (DSPC) Networks Show the Interaction That Existed Among Metabolites in ICP Patients

**FIGURE 4:**
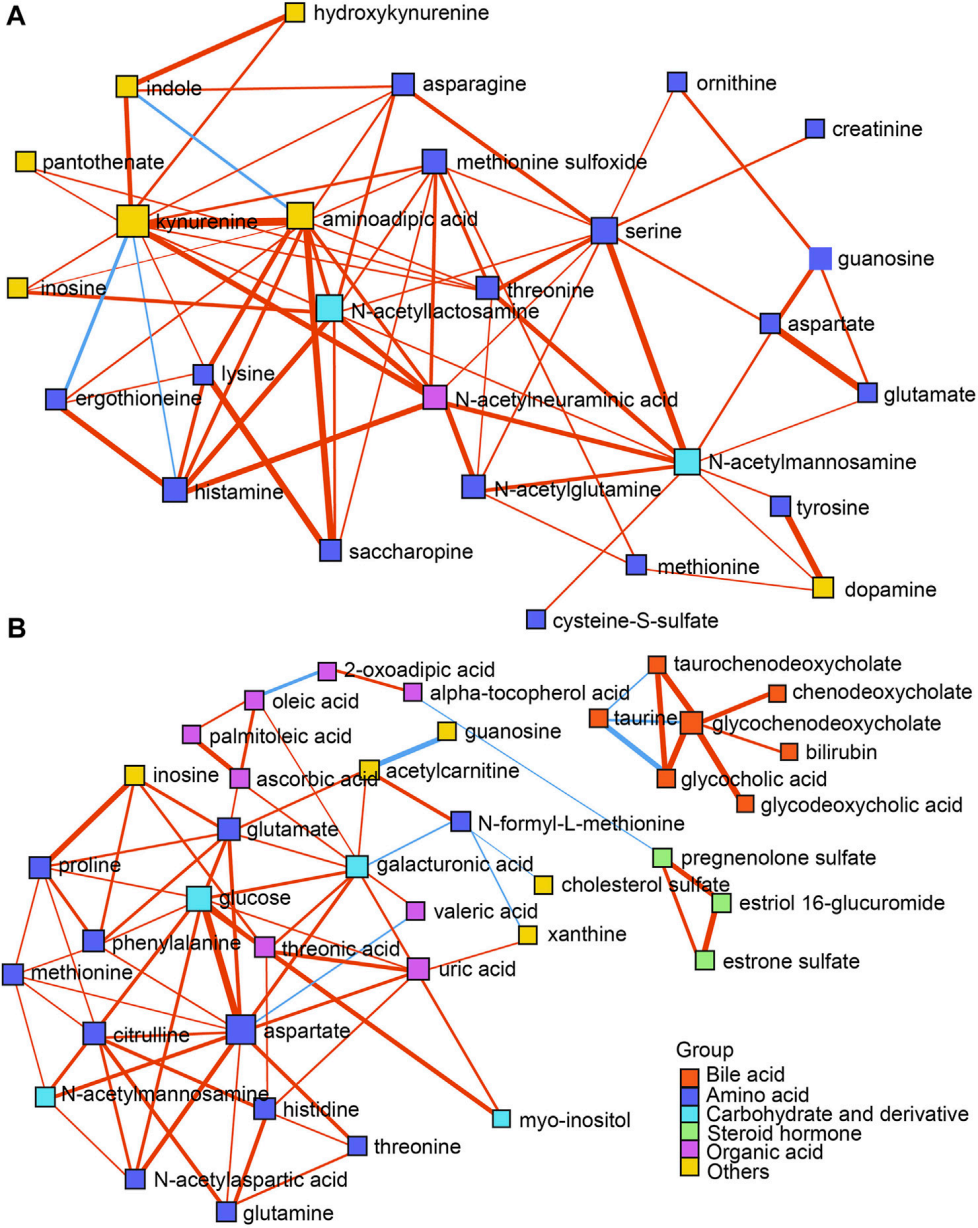
**(A,B)** DSPC network analysis of differential metabolites in both the ESI+ and ESI- modes, respectively. Nodes represent metabolites, edges represent the partial correlation of DSPC between two metabolites after conditioning on all other metabolites, width of edges represent the partial correlation coefficient strengths. Differential colors represent the classification of the metabolites. The red and blue lines indicate positive and negative correlations between two metabolites, respectively.

DSPC networks were constructed using all the differential metabolites to explore the potential mutual regulation among metabolites according to reported methods (Basu et al., 2017). As shown in Figures 4A,B, dense interactions among amino acids and their derivatives, organic acids, steroid hormones, as well as carbohydrates occurred in both the ESI+ and ESI- modes. Bile acids showed steady and independent partial correlations in the networks (Figure 4B). Kynurenine, *N*-acetyllactosamine, aminoadipic acid, and serine represented main hubs with dense interactions in the ESI + mode (Figure 4A), while glucose and aspartate represented the primary hubs with intensive interactions in the ESI- mode (Figure 4B), respectively. Notably, kynurenine negatively correlated with ergothioneine and histamine. Indole showed a negative association with aminoadipic acid in the ESI + mode (Figure 4A). The negative interactions between taurine and glycocholic acid, glycochenodeoxycholate, and taurochenodeoxycholate were found in the ESI- mode (Figure 4B). Several sporadic negative correlations including acetylcarnitine and guanosine, aspartate and valeric acid, oleic acid and 2-oxoadipic acid, *α*-tocopherol acid and pregnenolone sulfate, *N*-formyl-L-methionine and galacturonic acid, as well as xanthine and cholesterol sulfate were also exhibited (Figure 4B).

### Enrichment Analysis of Metabolic Pathways

**FIGURE 5:**
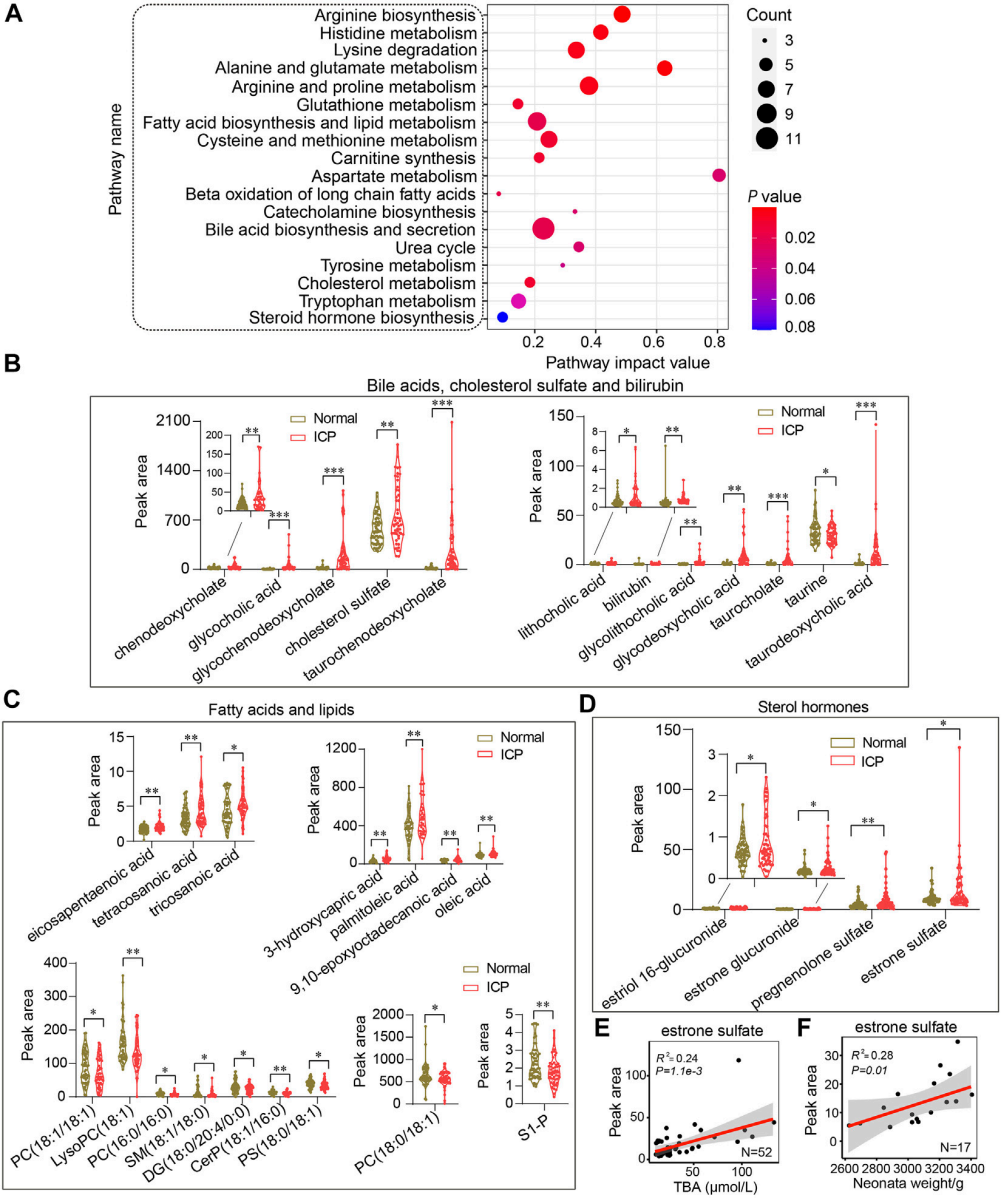
System-wide analysis of the metabolic pathways of ICP patients. **(A)** Metabolic pathway analysis of differential metabolites by using MetaboAnalyst. The size of the nodes shows the number of matched metabolites, the color of the nodes represents *p* value of the enrichment analysis. **(B–D)** Quantitative analysis of bile acids, cholesterol sulfate, bilirubin; fatty acids, lipids, and sterol hormones in the two groups, respectively. The Mann–Whitney *U* test is performed, ^*^ *p* < 0.05, ^**^ *p* < 0.01 vs. controls. **(E,F)** Pearson correlation analysis of estrone sulfate with TBA and neonatal weights in ICP patients, respectively. The 95% confidence interval for the linear regression is represented by the gray area.

A pathway analysis of differential metabolites was performed using MetaboAnalyst 4.0 (http://www.metaboanalyst.ca) (Chong et al., 2018). Parameters relating to the pathway analysis were set according to previous protocols (Chong et al., 2018; Chang et al., 2020). Pathway impact values were obtained by analyzing the cumulative percentage upon matched metabolites. Accordingly, the top 18 pathways were mapped into the Kyoto Encyclopedia of Genes and Genomes (KEGG) pathways (Figure 5A). Bile acid biosynthesis and secretion presented the highest enrichment ratio among all the pathways. The metabolites of bile acid biosynthesis and secretion showed a sharp increase except for taurine in the ICP group (Figure 5B). Noticeably, among all the pathways, arginine biosynthesis displayed the lowest *p* value (*p* = 1.5 × 10^–5^), whereas aspartate metabolism displayed the highest pathway impact with a value of 0.80 (Figure 5A). Correspondingly, in the pathway of fatty acid and lipid metabolism, the levels of the fatty acids were increased while the lipid levels were decreased in ICP patients (Figure 5C). Of note, two pathways relevant to mitochondrial oxidation, including carnitine synthesis and beta oxidation of long chain fatty acids displayed prominent changes in the ICP group (Figure 5A). Glutathione and cholesterol metabolisms showed moderate changes. Although the steroid hormone biosynthesis exhibited the lowest pathway impact (value = 0.08) (Figure 5A), the metabolites related to steroid hormones including estriol 16-glucuronide, estrone glucuronide, pregnenolone sulfate, and estrone sulfate displayed a dramatic increase in the ICP group (Figure 5D). Moreover, system-wide pathway disturbances were perceived in ICP patients. Of note, many pregnancy-related metabolites are implicated in various types of disease processes based on the metabolite set enrichment overview analysis, including dysphoric disorder, neonatal intrahepatic cholestasis, ornithine transcarbamylase deficiency, biliary atresia, etc. (Supplementary Figure S4).

### Bile Acid Levels are Relevant to MSAF in ICP Patients

**FIGURE 6:**
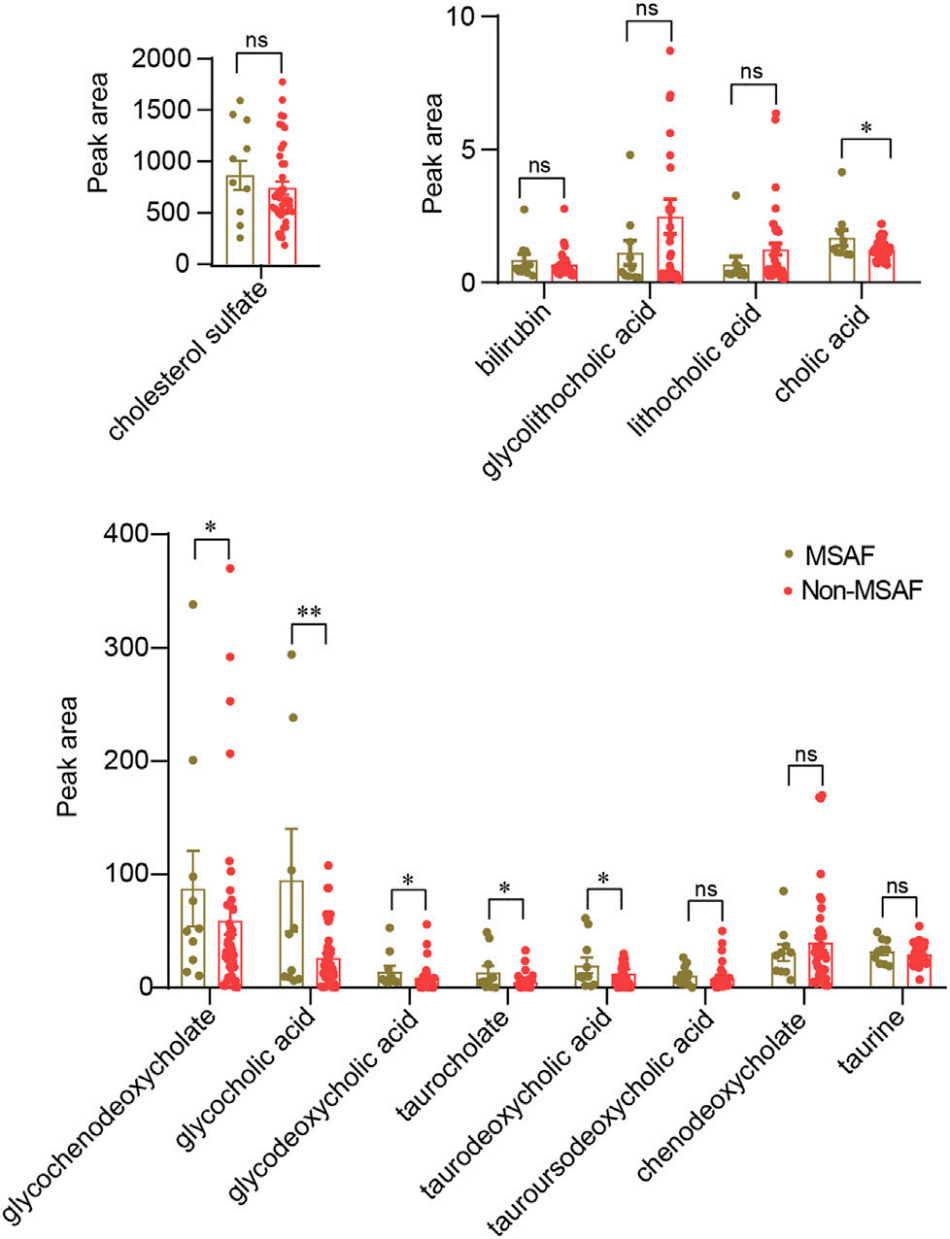
Quantitative analysis of the bile acid levels in the MSAF group and the non-MSAF group. Values representing the mean ± SEM, *p* values are presented with ^*^ *p* < 0.05, ^**^ *p* < 0.01 vs. controls, respectively. ns, non-significant; MSAF, meconium-stained amniotic fluid; Non-MSAF, Non-meconium-stained amniotic fluid.

As shown in Table 2, TBA, AST, ALT, TBIL, and DBIL levels were significantly altered in ICP patients with MSAF compared with those without MSAF (*p* < 0.05). There was no statistical difference in maternal age, the levels of BMI, ALP, GGT, ALB, TP, and GLB between the two groups. The levels of cholic acid, glycochenodeoxycholate, glycocholic acid, glycodeoxycholic acid, taurocholate, and taurodeoxycholic acid were significantly increased in the MSAF group, while the levels of cholesterol sulfate, tauroursodeoxycholic acid, and taurine showed an upward tendency with no statistical significance (Figure 6). Moreover, the level of bilirubin was significantly elevated in the MSAF group compared with the non-MASF group (Figure 6).

**TABLE 2:** Characteristics of ICP patients with MSAF and without MSAF.

Baseline information	Non-MSAF (*n* = 42)	MSAF (*n* = 10)	*p* value
Maternal age (y)^a^	30.1 ± 3.01	30.8 ± 2.52	0.491
BMI (kg/m^2^)^a^	26.17 ± 0.47	26.78 ± 1.64	0.453
TBA (μmol/L)^b^	22.05 (15.1–30.4)	59.75 (15.28–104.05)	<0.05
AST (U/L)^b^	36 (18–170.5)	163 (80.5–234.5)	<0.05
ALT (U/L)^b^	56 (12–329)	242.5 (152–275.5)	<0.05
ALP (U/L)^b^	217 (125–290)	262 (179.75–335.5)	0.160
GGT (U/L)^b^	30 (9–57.5)	39 (24–96.5)	0.158
ALB (g/L)^b^	34.1 (32.2–36.45)	32 (28.75–34.55)	0.07
TBIL (μmol/L)^b^	9.7 (7.65–11)	13.55 (7.93–24.45)	<0.05
DBIL (μmol/L)^b^	1.7 (1.05–2.8)	5.05 (2.23–12.4)	<0.01
TP (g/L)^b^	66.7 (63.25–69.75)	63.25 (57.3–70.6)	0.361
GLB (g/L)^b^	31.8 (30.2–33.45)	32.3 (27.15–36.68)	0.968

a Values are expressed as mean ± SEM (Students *t*-test).

b Values are expressed as median (interquartile range).

### The Level of Estrone Sulfate is Correlated with the Levels of TBAs and Neonatal Weights

It has been reported that estrogen and progesterone contribute to ICP development (Pařízek et al., 2015). Decreased steroid hormone levels in circulation have been found in patients with ICP (Leslie et al., 2000). The levels of the metabolites of estrogen and progesterone including estriol 16-glucuronide, estrone glucuronide, estrone sulfate, and pregnenolone sulfate were increased in ICP patients (Figure 5D). The Pearson correlation analysis shows that estrone sulfate levels were positively correlated with TBA levels and neonatal weights (*R* ^2^ = 0.24, *p* = 1.1e-3 and *R* ^2^ = 0.28, *p* = 0.01, respectively) (Figures 5E,F). Other metabolites of steroid hormones have no correlation with TBA levels or neonatal weights (*R* ^2^ <0.1) (Supplementary Figures S5A,B).

### Bile Acid Levels are Linked with Neonatal Weights

**FIGURE 7:**
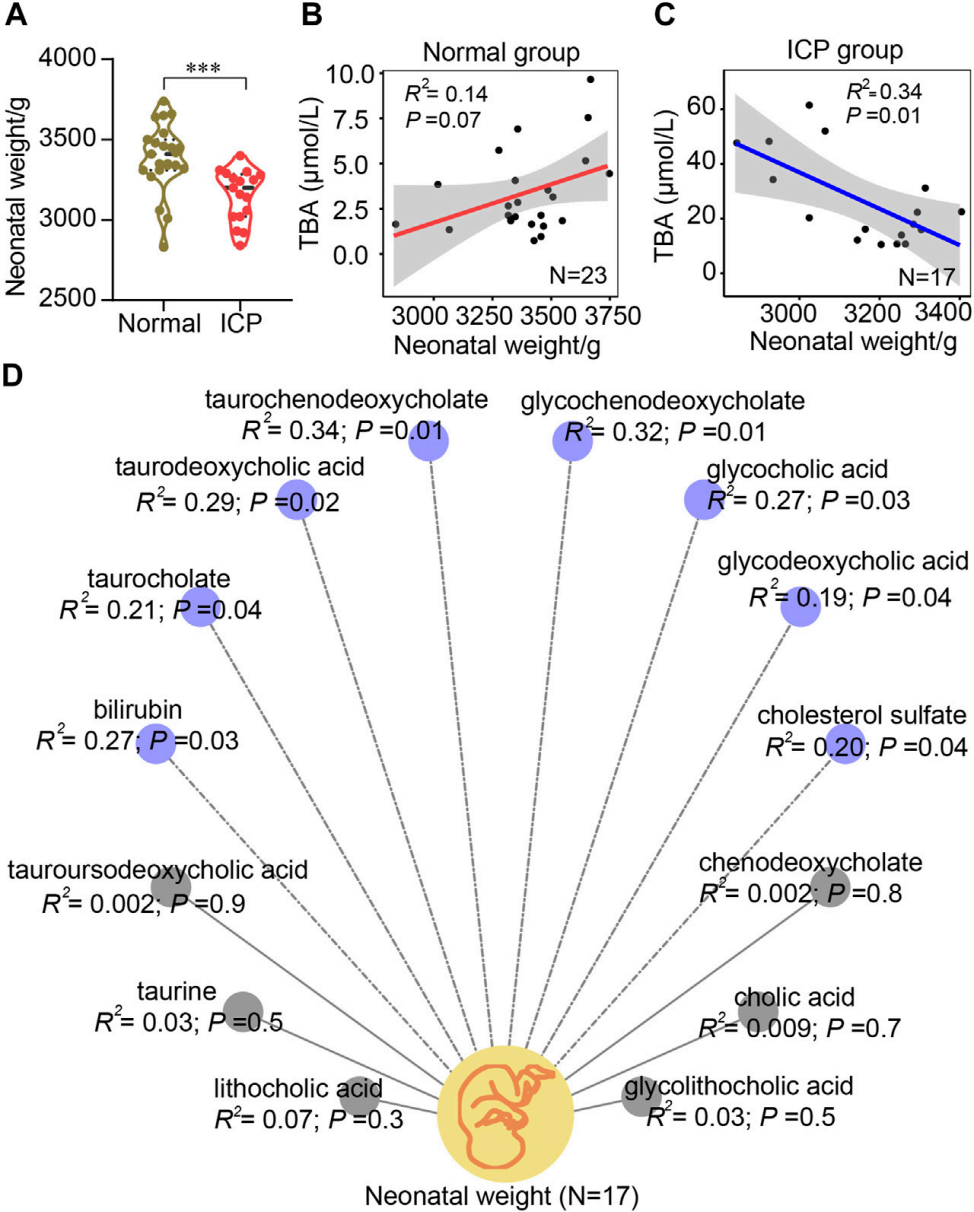
Pearson correlation analysis of bile acids and neonatal birthweights. **(A)** Quantitative analysis of neonatal weights in natural delivery control and the ICP group. Values representing the mean ± SEM, *p* values are presented with ^***^ *p* < 0.001 vs. controls. **(B,C)** Correlation analysis of bile acids and neonatal weights in the two groups, respectively. **(D)** Correlation analysis of each bile acid and neonatal weight in ICP patients with a normal delivery according to the mass spectrometric data, respectively. Blue (dotted lines) and gray (solid lines) circles represent negative and no correlations, respectively. The 95% confidence interval for the linear regression is represented by the gray area.

Neonatal weights in ICP patients at a term were significantly lower compared to those in controls (Figure 7A). No significant correlation between TBA level and neonatal weight was displayed in the normal group (*R* ^2^ = 0.14, *p* = 0.07); in contrast, a significant negative correlation was found in the ICP group (*R* ^2^ = 0.34, *p* = 0.01) (Figures 7B,C). A negative correlation between neonatal weights and six bile acids, including glycochenodeoxycholate, glycocholic acid, glycodeoxycholic acid, taurochenodeoxycholate, taurocholate, and taurodeoxycholic acid, was found in ICP patients (Figure 7D). Among them, taurochenodeoxycholate showed the highest (*R* ^2^ = 0.34, *p* = 0.01), and glycodeoxycholic acid displayed the lowest (*R* ^2^ = 0.19, *p* = 0.04) correlation coefficients. Bilirubin and cholesterol sulfate levels were negatively associated with neonatal weights (*R* ^2^ = 0.27, *p* = 0.03, and *R* ^2^ = 0.20, *p* = 0.04, respectively) (Figure 7D) while chenodeoxycholate, tauroursodeoxycholic acid, cholic acid, taurine, lithocholic acid, and glycolithocholic acid demonstrated no significant correlation (*R* ^2^ < 0.1) (Figure 7D). Elevated bile acid levels have no significant correlation with gestational age (Supplementary Figure S6).

### Tryptophan Metabolism is Related to Elevated TBA Levels

**FIGURE 8:**
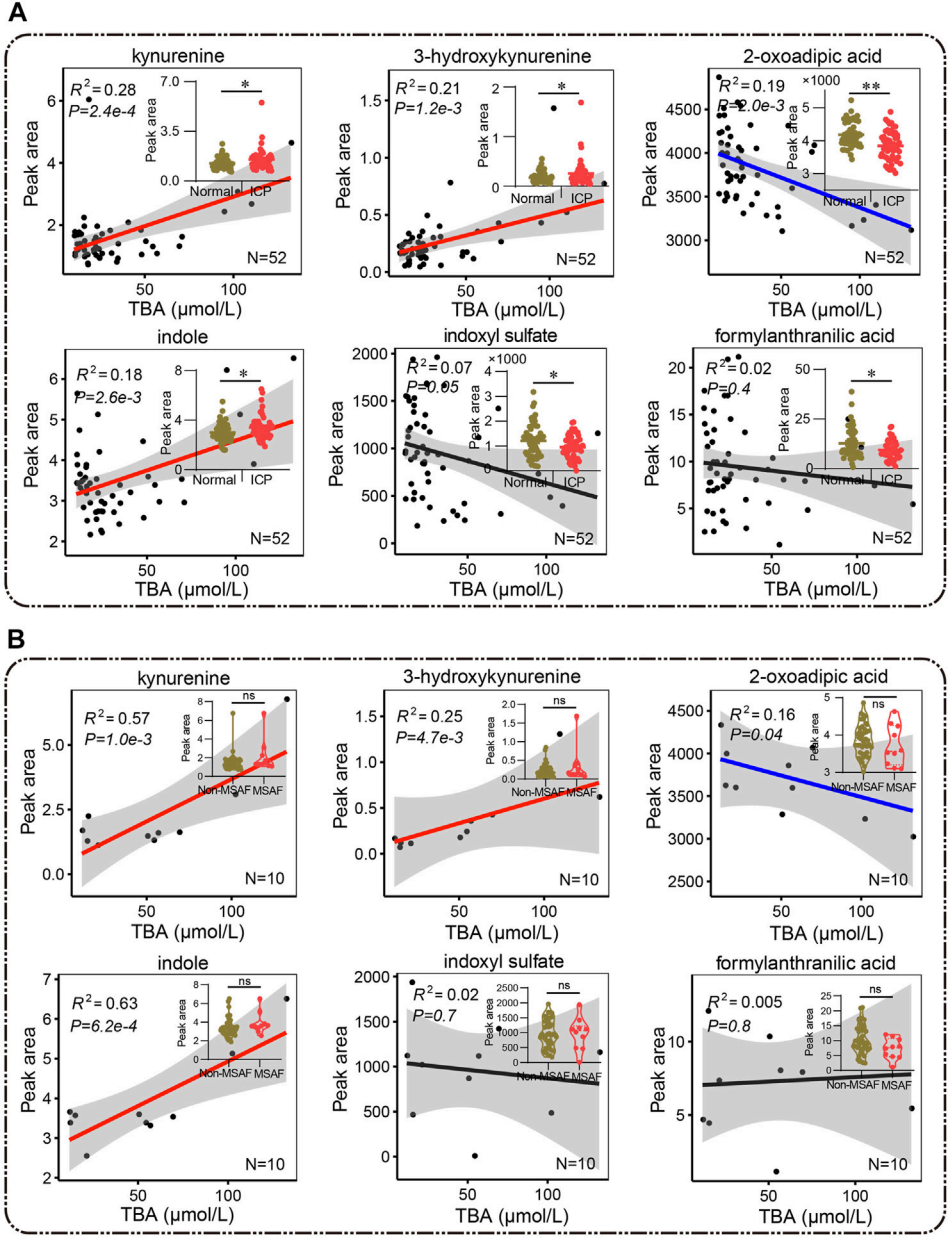
**(A,B)** Pearson correlation analysis between metabolites of the tryptophan pathway and bile acid. Red and blue lines represent positive and negative correlations, and the black lines show no correlation, respectively. **(A)** The Mann–Whitney *U* test and **(B)** the unpaired Student’s *t*-test are performed respectively, ^*^ *p* < 0.05, ^**^ *p* < 0.01 vs. controls. ns, non-significant; MSAF, meconium-stained amniotic fluid; Non-MSAF, Non-meconium-stained amniotic fluid.

Bile acid and tryptophan metabolism disorder has been implicated in BPA-induced maternal and fetal metabolic diseases (Susiarjo et al., 2017). Tryptophan catabolites including kynurenine, 3-hydroxykynurenine, indole, indoxyl sulfate, formylanthranilate, and 2-oxoadipic acid were significantly changed in the ICP group, suggesting a disturbed tryptophan metabolism (Figure 8A). A positive correlation between kynurenine, 3-hydroxykynurenine and indole, and elevated bile acid levels (*R* ^2^ = 0.28, *p* = 2.4e-4; *R* ^2^ = 0.21, *p* = 1.2e-3; and *R* ^2^ = 0.18, *p* = 2.6e-3) was identified, while a negative correlation was noticed between 2-oxoadipic acid and bile acid levels (*R* ^2^ = 0.19, *p* = 2.0e-3), respectively (Figure 8A). Notably, kynurenine and indole showed a highly positive correlation with bile acid levels (*R* ^2^ = 0.57, *p* = 1.0e-3, and *R* ^2^ = 0.63, *p* = 6.2e-4, respectively) while no statistical difference of these two metabolites were found in the patients with MSAF compared to patients without MSAF (Figure 8B). Typically, 3-hydroxykynurenine and 2-oxoadipic acids exhibit moderately positive and negative correlations with bile acid levels in ICP patients with MSAF (*R* ^2^ = 0.25, *p* = 4.7e-3 and *R* ^2^ = 0.16, *p* = 0.04), respectively (Figure 8B).

### ICP Patients Exhibit an Underlying Low Antioxidant Capacity or/and High Oxidative Stress

**FIGURE 9:**
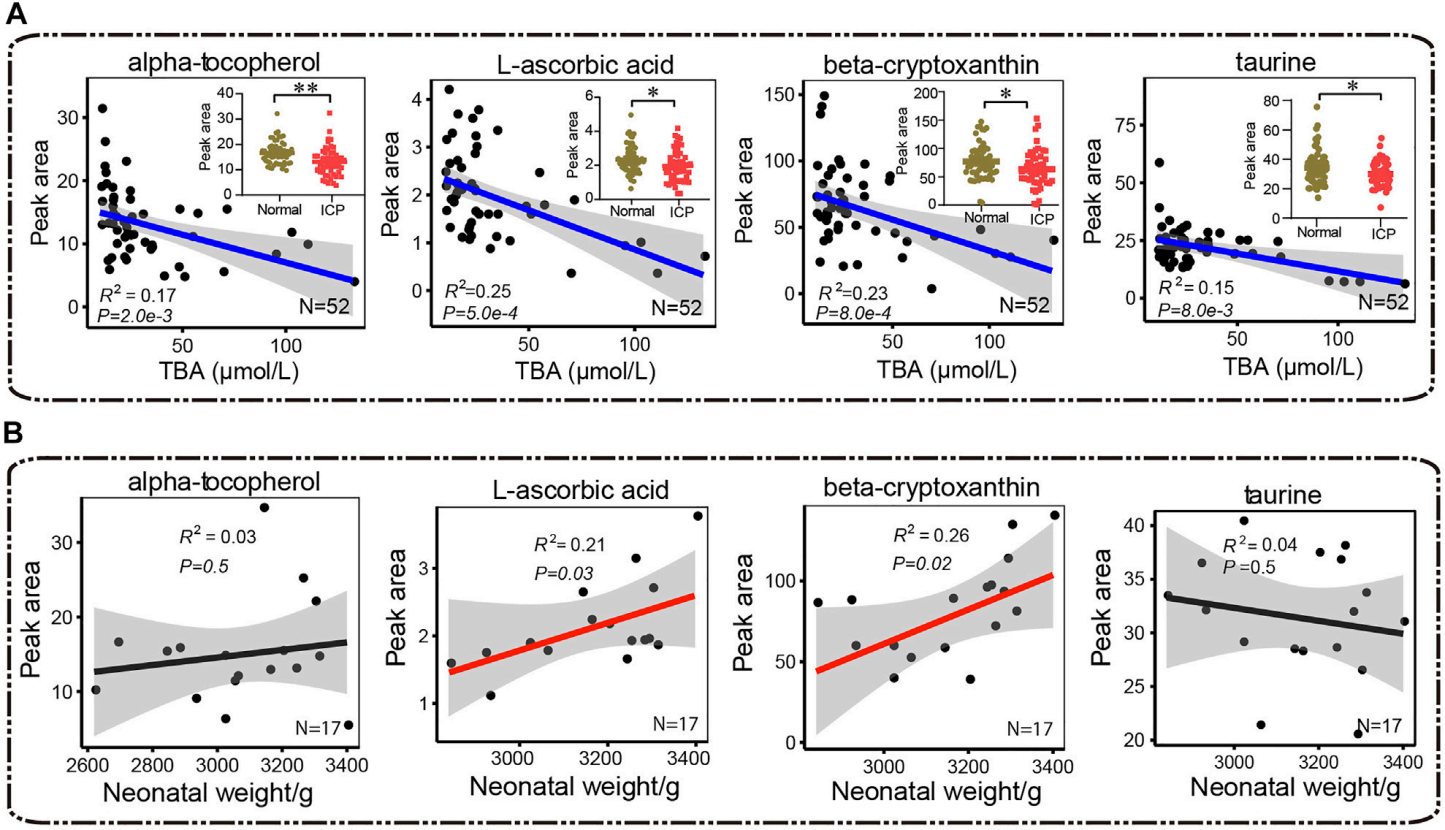
Pearson correlation analysis between antioxidants (*α*-tocopherol, L-ascorbic acid, *β*-cryptoxanthin, and taurine) and **(A)** total bile acids and **(B)** neonatal weights, respectively. Red and blue lines represent positive and negative correlations, and the black lines show no correlation, respectively. The 95% confidence interval for the linear regression is represented by the gray area. The Mann–Whitney *U* test is performed, ^*^ *p* < 0.05, ^**^ *p* < 0.01 vs. control, respectively.

Alpha-tocopherol, L-ascorbic acid, beta-cryptoxanthin, and taurine are able to inhibit lipid peroxidation and scavenge free radicals (Burri et al., 2016; Esrefoglu et al., 2016; Niu et al., 2018; Di Vincenzo et al., 2019). The ICP group showed significantly lower levels in *α*-tocopherol, L-ascorbic acid, beta-cryptoxanthin, and taurine compared with the control group (Figure 9A). In addition, *α-*tocopherol, L-ascorbic acid, beta-cryptoxanthin, and taurine levels were inversely correlated with TBA levels (*R* ^2^ = 0.17, *p* = 2.0e-3; *R* ^2^ = 0.25, *p* = 5.0e-4; *R* ^2^ = 0.23, *p* = 8.0e-4, and *R* ^2^ = 0.15, *p* = 8.0e-3, respectively) (Figure 9A). The levels of L-ascorbic acid and beta-cryptoxanthin were positively correlated with neonatal weights (*R* ^2^ = 0.21, *p* = 0.03, and *R* ^2^ = 0.26, *p* = 0.02). The correlation of *α*-tocopherol and taurine with neonatal weights showed no statistical significance (*R* ^2^ = 0.03, *p* = 0.5, and *R* ^2^ = 0.04, *p* = 0.5, respectively) (Figure 9B).

### The Levels of LCSFAs are Correlated with the Levels of TBAs

The accumulation of LCSFAs in circulation is deemed as potentially toxic constituents (Rial et al., 2010). As shown in Figures 5A, C, a significant increase of free fatty acids was found in ICP patients. The levels of tricosanoic acid, tetracosanoic acid, and 3-hydroxycapric acid were positively correlated to TBA levels (*R* ^2^ = 0.27, *p* = 8.06e-5; *R* ^2^ = 0.23, *p* = 6.52e-4, and *R* ^2^ = 0.19, *p* = 1.24e-4), respectively (Supplementary Figure S7A). No correlation of poly-unsaturated fatty acid (PUFA), including palmitoleic acid, oleic acid, and eicosapentaenoic acid with TBAs was observed in ICP patients (*R* ^2^ < 0.1) (Supplementary Figure S7A). No correlation was found between the levels of LCSFA or PUFA with newborn weights (*R* ^2^ < 0.1) (Supplementary Figure S7B).

### Newborn Body Weights Are Associated with the Levels of Some Amino Acids.

Lysine and asparagine levels showed positive correlations with newborn weights (*R* ^2^ = 0.23, *p* = 0.04, and *R* ^2^ = 0.22, *p* = 0.04, respectively) (Supplementary Figure S8). The level of ergothioneine, a natural metabolite of histidine, was positively correlated with newborn weights (*R* ^2^ = 0.25, *p* = 0.04) (Supplementary Figure S8). Positive correlations of newborn weights with guanosine and N-acetyllactosamine levels were also found (*R* ^2^ = 0.25, *p* = 0.03, and *R* ^2^ = 0.22, *p* = 0.04, respectively).

## Discussion

**FIGURE 10:**
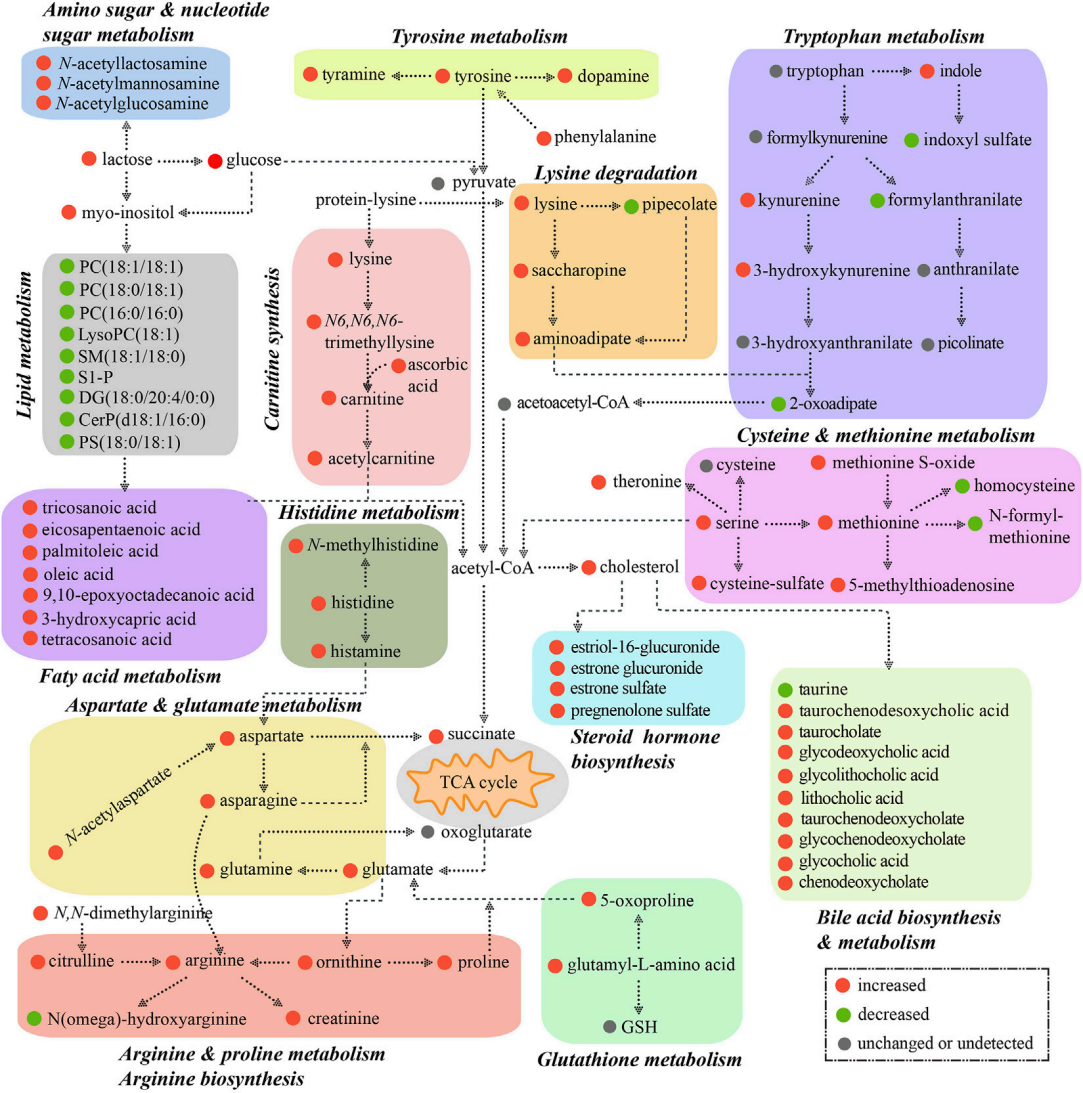
Metabolic pathway map associated with patients of ICP. Increased and decreased metabolites are highlighted in red and green, respectively. Gray color represents no significant change or undetected. The background colors indicate different metabolic pathways.

In this study, we have shown circulatory metabolomic profiles in ICP patients by using untargeted metabolomics, affirmed some pregnancy-related metabolites which are disclosed by some studies (Liang et al., 2020), such as estriol 16-glucuronide, estrone sulfate, pregnenolone sulfate, etc., and revealed that multiple metabolic pathways were disturbed in ICP patients and the levels of a number of metabolites were correlated with TBA levels and neonatal weights. We also strived to portray metabolic pathway networks based on the interaction among input metabolites and the KEGG database so as to understand the overall changes of the blood metabolome in ICP patients. Notably, all metabolic pathways are interrelated and constitute a complicated metabolic network (Figure 10).

Dong et al. (2021) have recently shown the placental metabolomic profile in ICP patients, and demonstrated that fatty acid metabolism, primary bile acid synthesis, tryptophan metabolism, and arginine and proline metabolism are enriched. Interestingly, our study about the maternal circulatory metabolomic profile has revealed the enriched pathways including bile acid biosynthesis and secretion, fatty acid biosynthesis and lipid metabolism, and tryptophan metabolism and arginine and proline metabolism in ICP patients. It suggests that a circulatory metabolomic profile can, at least in part, reflect the metabolic state of the placenta of an ICP patient. Additionally, we found that the pathways of carnitine synthesis and *β-*oxidation of long chain fatty acids were prominently changed in the circulation of the ICP group, which is consistent with Dong et al’s study where they have shown that the metabolites and proteins related to carnitine synthesis and *β*-oxidation of long chain fatty acids are significantly changed in ICP placentas. Of note, the alteration in carnitine synthesis and long chain fatty acid *β-*oxidation is associated with an abnormal mitochondrial function.

We enriched the pathway of glutathione metabolism in ICP patients, suggesting that oxidiative stress occurs in ICP patients. Moreover, the levels of a number of metabolites that are relevant to the antioxidant system, such as *α*-tocopherol, taurine, L-ascorbic acid, and *β-*cryptoxanthin, were markedly reduced in ICP patients. Taurine is an important free radical scavenger and an antioxidant in organisms. The deficiency of taurine during pregnancy leads to an elevated oxidative stress in the mother, and results in a premature delivery and low birth weight in the fetus (Lerdweeraphon et al., 2013). L-ascorbic acid inhibits oxidation of membrane phospholipids, and maintains the cellular antioxidant system (Esrefoglu et al., 2016). *β-*cryptoxanthin can act as an antioxidant to prevent free radical damage in the DNA (Burri et al., 2016). *α*-Tocopherol (vitamin E) is known to be a powerful antioxidant. The level of *α*-Tocopherol in maternal circulation is positively associated with fetal growth (Scholl et al., 2006). We also found that L-ascorbic acid and *β-*cryptoxanthin levels were positively correlated with neonatal weights in ICP patients, implying that abnormal levels of these metabolites increase the risk of adverse pregnancy outcomes.

In the present study, a significant negative correlation between six bile acids and neonatal weights was found. However, there was no small-for the gestational age case in the participants. Whether elevated bile acid levels are potential adverse factors for fetal growth remains to be confirmed in a large scale of participants in a future study. Kawakita et al. (2015) have shown that there is no significant difference in hyperbilirubinemia and neonatal weights among ICP patients with mild (10–39.9 μmol/L), moderate (40–99.9 μmol/L), and severe TBA levels (≥100 μmol/L). However, their study lacks the group of healthy pregnant women. Our data showed that hyperbilirubinemia occurred in ICP patients, and that the bilirubin level was negatively correlated with neonatal weights. Six ICP patients with hyperbilirubinemia had TBA levels >34 μmol/L (75th centile in our data), while four of which had MSAF (account for 66.7% of hyperbilirubinemia), suggesting that hyperbilirubinemia is associated with an increased risk of MSAF. High TBA levels can cause vasoconstriction in placental chorionic veins, thereby leading to fetal hypoxia and MSAF (Sepúlveda et al., 1991). TBA level> 40 μmol/L is regarded as a predictor of an adverse neonatal outcome in ICP (Kawakita et al., 2015). We found that 50% of ICP patients with TBA>40 μmol/L had MSAF, while only 10% of ICP patients with TBA<40 μmol/L had MSAF, suggesting that patients with a TBA level>40 μmol/L have an increased risk of MSAF.

It is known that steroid hormones, mainly estrogen and progesterone, play crucial roles in the pathogenesis of ICP. Previous studies have shown that an elevated estrogen level can cause hepatocellular cholestasis, which may be correlated with the abnormal expression of hepatic biliary proteins and the bile acid transporter bile salt export pump (Song et al., 2014; Chen et al., 2015). Several estrogen and progesterone metabolites induce trans-inhibition of the bile salt export pump and subsequently lead to an accumulation of bile acids (Vallejo et al., 2006). These studies about the alternation in steroid hormones in ICP patients are varied (Leslie et al., 2000; Pařízek et al., 2015), which might be attributed to complicated effects of the hormones and ethnicity and environmental factors. Estrone sulfate is the most abundant circulating estrogen in pregnant women; it acts as a long-lived reservoir that could be converted as needed to the more active estradiol (Geyer et al., 2017). Pregnenolone sulfate is the precursor of progesterone and is transformed into progesterone by enzymes (Geyer et al., 2017). As a partial agonist of FXP, pregnenolone sulfate may be one of the potential factors linking progesterone and its metabolites to the ICP pathogenesis (Pařízek et al., 2016; Geyer et al., 2017). It is worth noting that our data reveal increments of both estrogen and progesterone in ICP. Nevertheless, estrone sulfate levels are not only positively associated with bile acids but also with neonatal weights, suggesting that an highly active estrone sulfate may be a more important factor in ICP development.

An increasing body of evidence has indicated that cross-talk between bile acids and gut bacteria has an important impact on host metabolism (Wahlström et al., 2016; Jia et al., 2018). The effects of gut bacteria on the tryptophan metabolism in humans have been studied extensively (Agus et al., 2018). Over 90% of tryptophan is a substrate in the kynurenine pathway. There are many metabolites, such as kynurenine, 3-hydroxykynurenine, and 2-oxoadipic in this pathway. Microbiota-driven tryptophan metabolites, such as indole and its intermediates, are the ligands of the aryl hydrocarbon receptor. They play critical roles in intestinal homeostasis by regulating inflammatory signals (Lamas et al., 2018; Natividad et al., 2018). Our data show that kynurenine, 3-hydroxykynurenine, 2-oxoadipic acid, and indole levels are associated with the bile acid levels. The level of valeric acid, a gut microbial specific metabolite, is decreased, whilst the level of pantothenate (vitamin B5), which is primarily produced by gut microbiota, is increased in ICP patients. These data suggest that an abnormal tryptophan metabolism may be connected with enteric dysbacteriosis in ICP patients.

The major limitations of this study are: 1) a relatively small size in samples may have impact on the accuracy of the correlation analysis; 2) the interactions between an abnormal tryptophan metabolism and gut microbiota, as well as the effects on adverse pregnancy outcomes should be further studied and verified; 3) metabolomic study is not absolutely quantified. Thus, a targeted metabolomics and absolute quantification study should be performed in the succedent works.

## Conclusion

This study demonstrates that multiple metabolic pathways are disturbed in ICP patients, and the levels of a number of metabolites are correlated with TBA levels and neonatal weights. It is also revealed that abnormal tryptophan metabolism, elevated estrone sulfate and LCSFA levels, and a low antioxidant capacity are relevant to increased bile acid levels. Our study highlights the characteristics of metabolomics in circulation and indicates that disturbed metabolic pathways are associated with adverse pregnancy outcomes in ICP patients.

## Data Availability

All raw data of mass spectrometry have been deposited to the MetaboLights public database (Accession number: MTBLS2627 accessed by link: https://www.ebi.ac.uk/metabolights).
