# Supplementary material for: Circulatory Metabolomics Reveals the Association of the Metabolites With Clinical Features in the Patients With Intrahepatic Cholestasis of Pregnancy
Source: Front Physiol. 2022 Jul 11;13:848508. doi: 10.3389/fphys.2022.848508 (PMC9309339; doi:10.3389/fphys.2022.848508)
Supplement: Supplementary file 1 [file DataSheet1.docx]

Supplementary Material

# Supplementary Figures


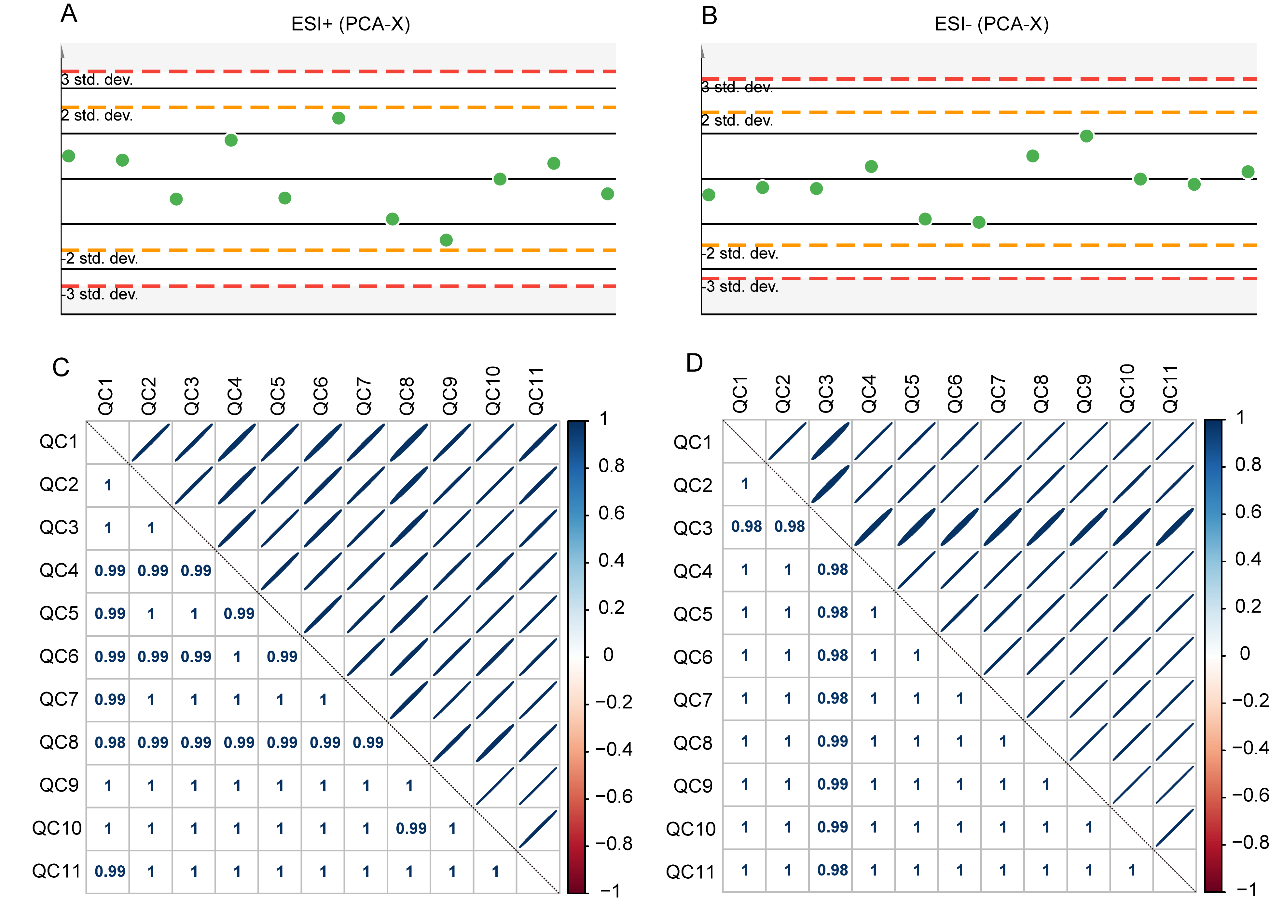


**Figure 1**. Data quality control analysis. PCA-X one-dimensional line plots from QC samples to assess the experiment reproducibility of control group and ICP group in ESI+ (A) and ESI− (B), respectively. Spearman’s correlation coefficients from QCs samples to assess the experiment reproducibility of control and ICP groups in ESI+ (C) and ESI− (D), respectively.


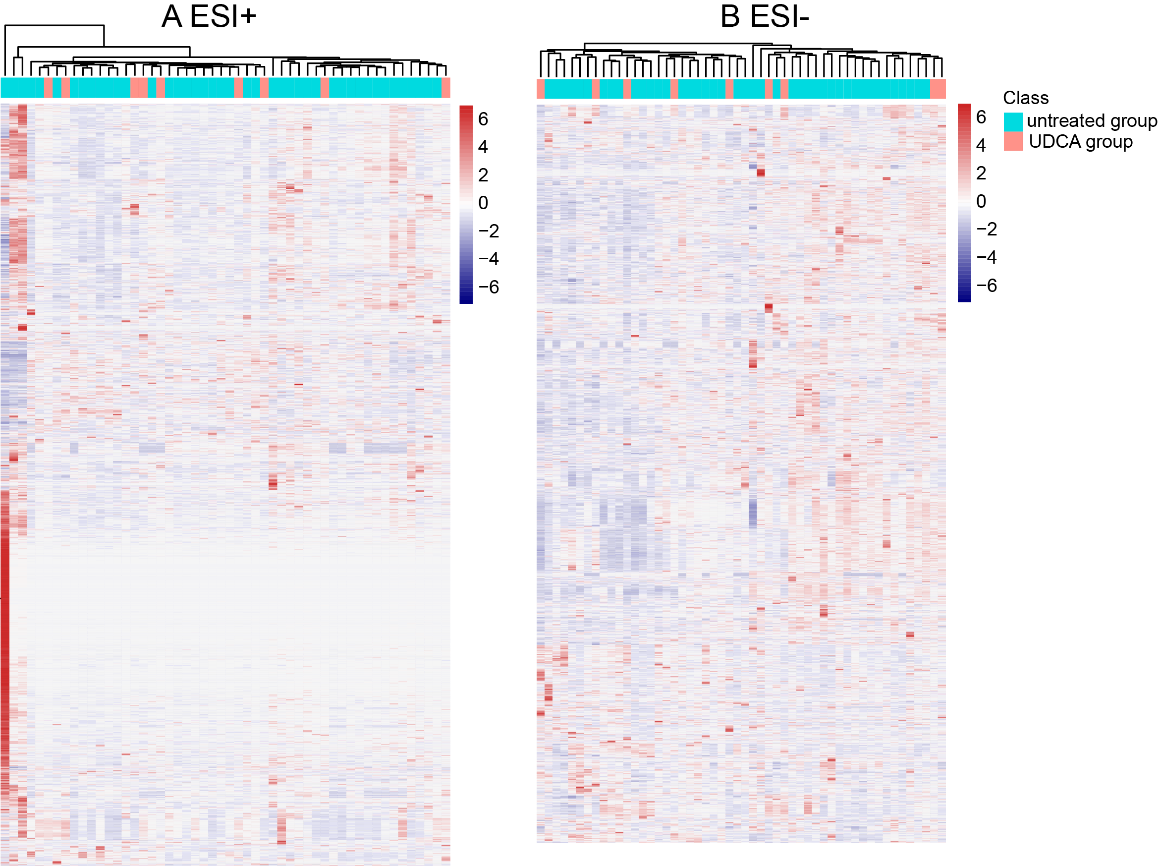


**Figure 2.** Heatmap visualizes the metabolites signal intensity of UDCA treated and untreated group in ESI+ and ESI- modes, respectively. Row represents metabolites and column represents samples.


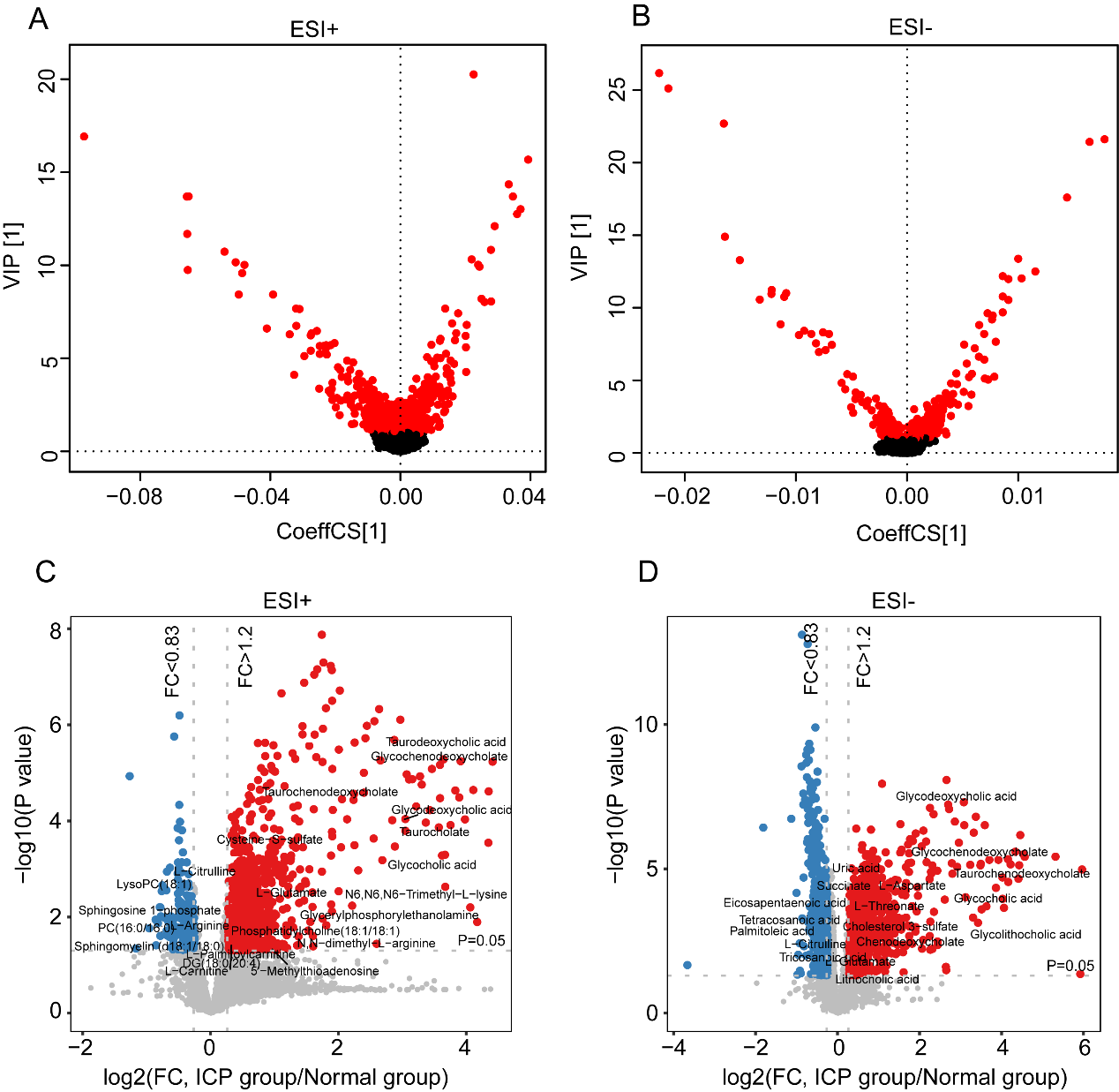


**Figure 3**. Analysis of diﬀerential metabolites between control and ICP groups. V-plots of control and ICP groups in ESI+ (A) and ESI– (B) modes, respectively. Red nodes represent corresponding variables with VIP > 1.0 in each group. Diﬀerential metabolites in control and ICP groups in ESI+(C) and ESI− (D) modes are screened by volcano plot respectively. Metabolites which showed fold changes greater than 1.2 or less than 0.83 and *P*<0.05 are regarded as increased and decreased and marked in red and blue, respectively. The gray dots are considered as no signiﬁcant change.


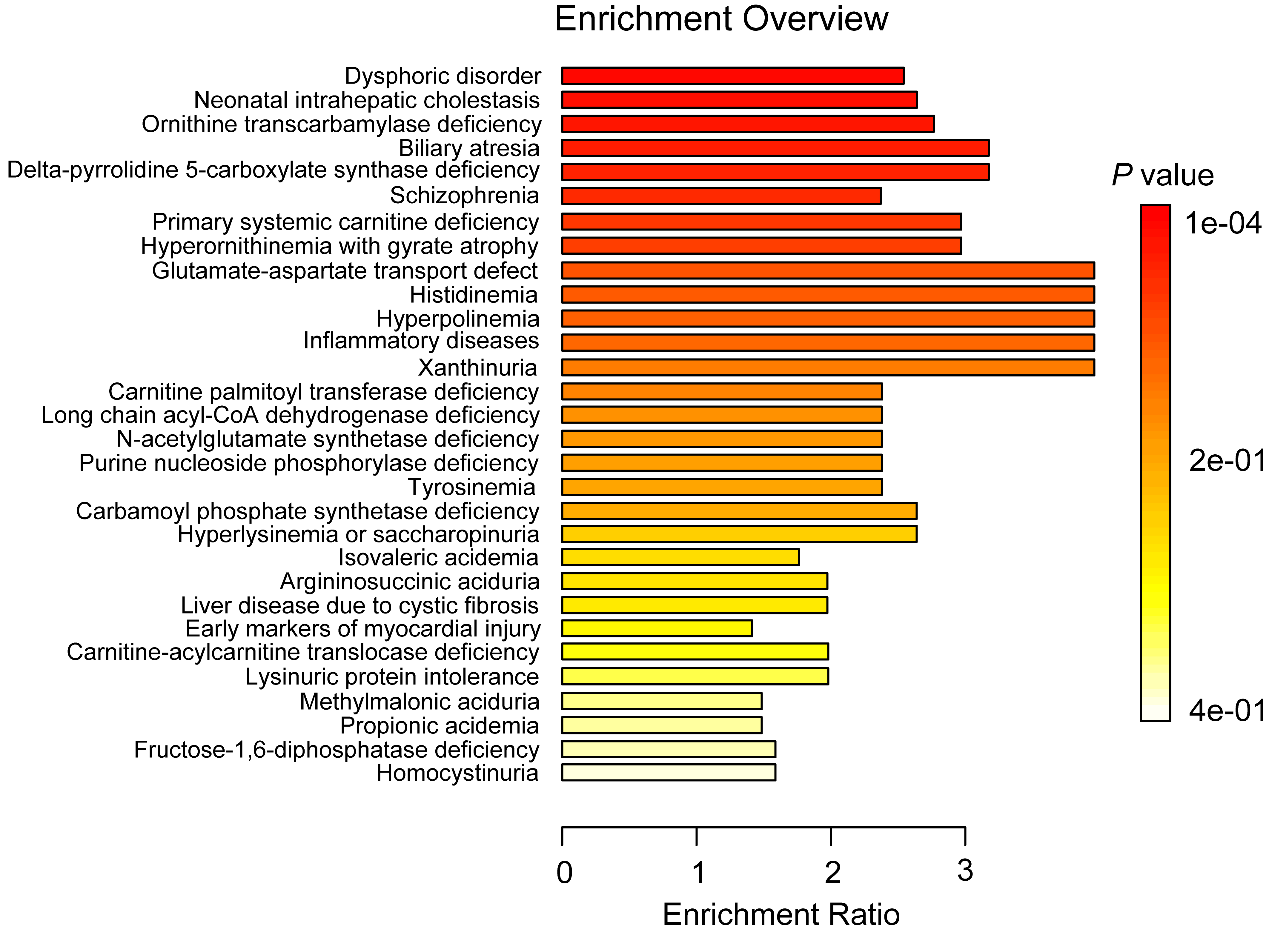


**Figure 4**. Enrichment of pathways related to intrahepatic cholestasis during pregnancy on the basis of differential metabolites by means of human disease database.


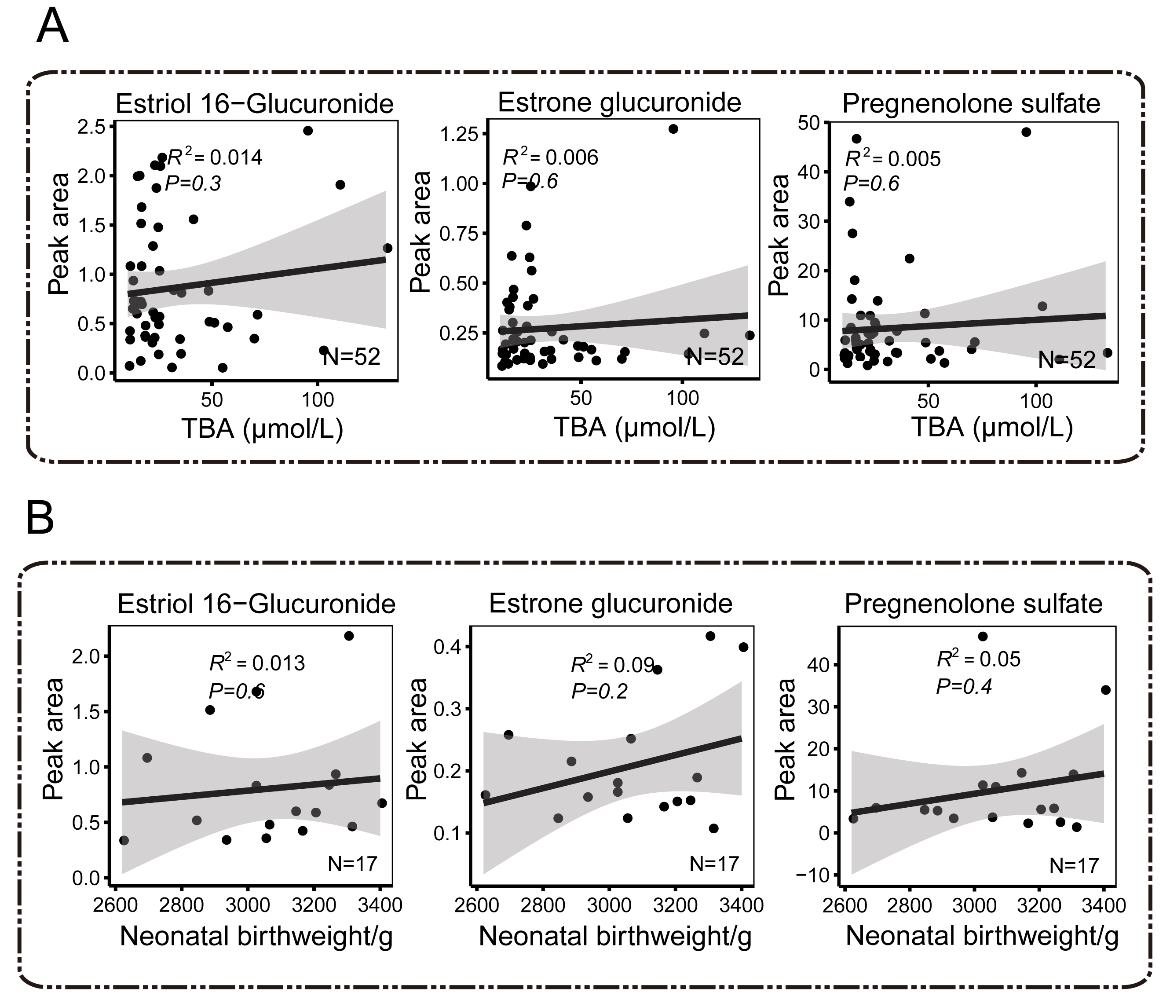


**Figure 5**. Correlation analysis of total bile acid, steroid hormones (A) and neonatal weights (B) in ICP patients, respectively. The 95% confidence interval for the linear regression is represented by the gray area.


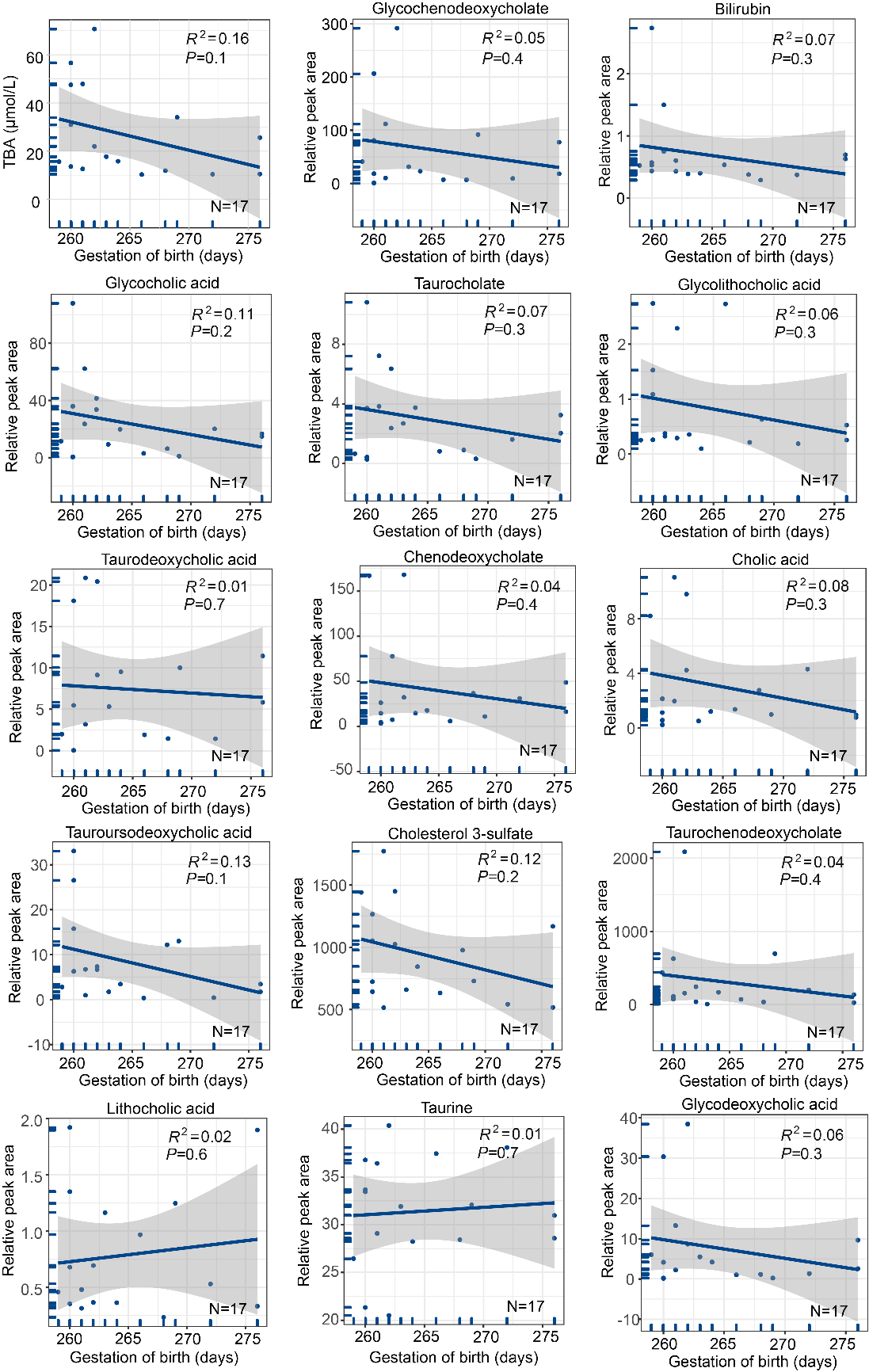


**Figure 6.** Pearson correlation analysis of bile acid and gestation of birth, respectively. The 95% confidence interval for the linear regression is represented by the gray area.


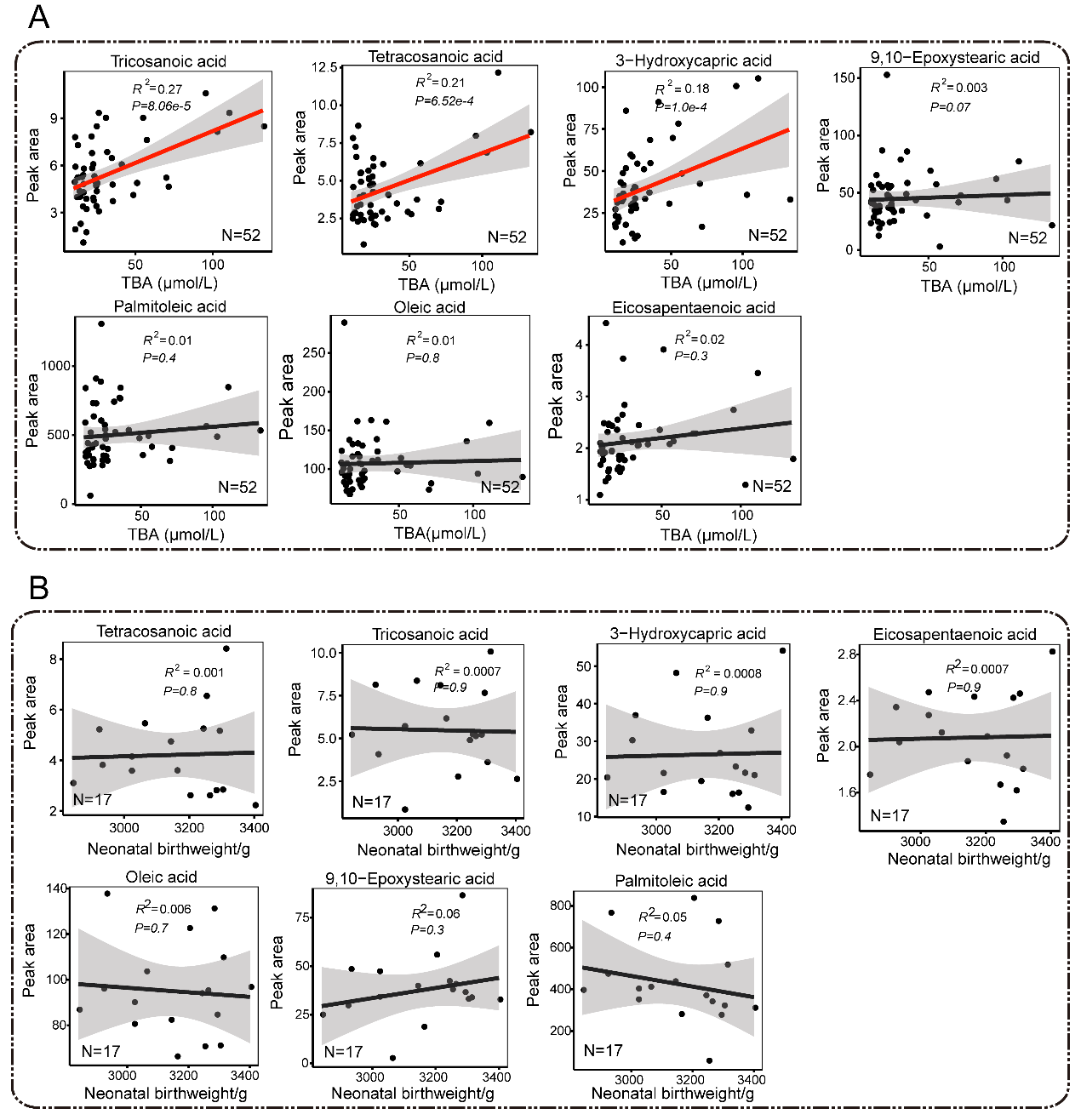


**Figure 7**. Correlation analysis of total bile acid, fatty acids (A) and neonatal weights (B) in ICP patients, respectively. Red lines represent positive correlation. The 95% confidence interval for the linear regression is represented by the gray area.


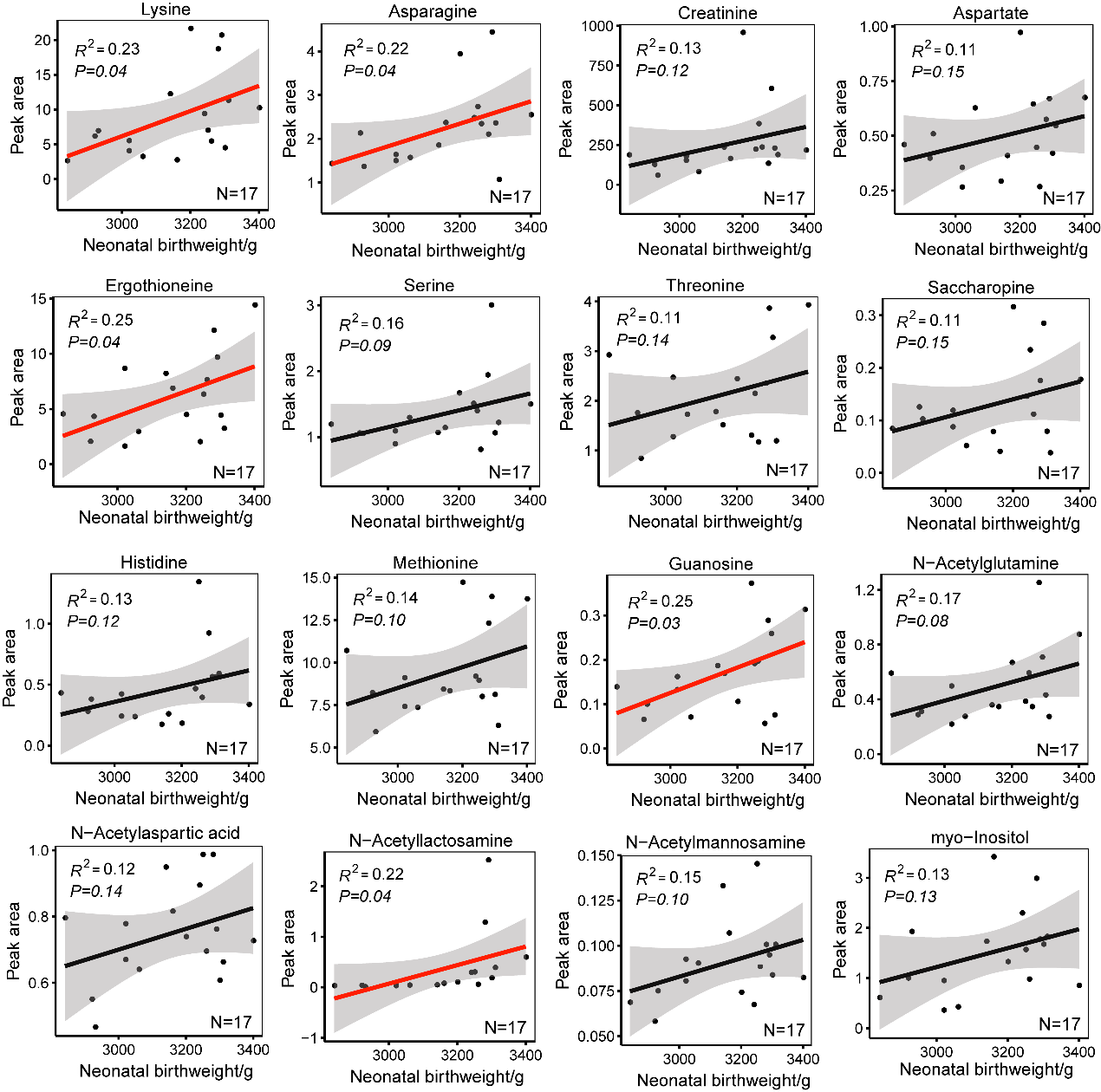


**Figure 8.** Pearson correlation analysis of amino acids, carbohydrates and neonatal weights. Red and black lines represent positive and no significantly correlation, respectively. The 95% confidence interval for the linear regression is represented by the gray area.

# Supplementary Table

Table 1 Altered metabolites of ICP group compared with control group

| Name | Adduct | Observed MS | Exact MS | MS error /ppm | MS2 score | VIP^*^ | RT(s) ^†^ | FC^‡^ | *P* value | ESI^§^ |
| --- | --- | --- | --- | --- | --- | --- | --- | --- | --- | --- |
| PC(18:1/18:1) | (M+H-H_2_O)^+^ | 768.58817 | 785.59346 | -6.7 | 0.9973 | 3.1 | 92.59 | 0.78 | 0.02 | + |
| LysoPC(18:1) | (M+Na)^+^ | 544.33848 | 521.34814 | -1.9 | 0.9724 | 5.1 | 189.21 | 0.81 | 0.003 | + |
| PC(18:0/18:1) | (M+Na)^+^ | 810.59934 | 787.60910 | -1.2 | 0.9999 | 6.0 | 141.34 | 0.82 | 0.04 | + |
| 3-Methylhistidine | (M+H)^+^ | 170.09169 | 169.08513 | 3.9 | 0.9938 | 1.8 | 374.28 | 1.46 | 0.02 | + |
| Creatinine | (M+H)^+^ | 114.06557 | 113.05891 | 5.9 | 0.9962 | 5.3 | 169.08 | 1.50 | 3.0×10^-4^ | + |
| Glycodeoxycholic acid | (M+NH_4_)^+^ | 467.34633 | 449.31412 | 7.2 | 0.9835 | 3.2 | 211.65 | 10.94 | 8.4×10^-6^ | + |
| Arginine | (M+H)^+^ | 175.11832 | 174.11168 | 3.8 | 0.9984 | 1.3 | 502.57 | 1.32 | 0.009 | + |
| Citrulline | (M+H)^+^ | 176.10219 | 175.09569 | 3.7 | 0.9847 | 1.2 | 387.89 | 1.32 | 0.002 | + |
| Glutamate | (M+H)^+^ | 147.05988 | 146.06914 | -6.3 | 0.9875 | 2.0 | 396.10 | 1.34 | 0.003 | + |
| Methionine | (M+H)^+^ | 150.0575 | 149.05105 | 4.3 | 0.9813 | 1.4 | 282.24 | 1.31 | 7.5×10^-5^ | + |
| Phenylalanine | (M+H)^+^ | 166.08588 | 165.07898 | 4.2 | 0.9747 | 2.9 | 255.30 | 1.20 | 0.01 | + |
| Pyroglutamic acid | (M+NH_4_)^+^ | 147.03609 | 129.04259 | -5.0 | 0.9976 | 1.9 | 370.18 | 1.21 | 0.02 | + |
| Palmitoylethanolamide | (M+H)^+^ | 300.28908 | 299.28243 | 2.2 | 0.9926 | 1.3 | 95.28 | 2.11 | 2.0×10^-4^ | + |
| PC(16:0/16:0) | (M-H+2Na)^+^ | 778.53638 | 733.56215 | -3.5 | 0.9920 | 1.1 | 102.36 | 0.75 | 0.01 | + |
| SM (d18:1/18:0) | (M+H)^+^ | 731.60428 | 730.59887 | 0.7 | 0.9999 | 1.2 | 199.88 | 0.58 | 0.02 | + |
| Taurocholate | (M+H)^+^ | 516.29822 | 515.29167 | 1.3 | 0.9800 | 2.6 | 206.82 | 10.24 | 1.0×10^-4^ | + |
| Taurodeoxycholic acid | (M+NH_4_)^+^ | 517.32889 | 499.29676 | 6.3 | 0.9924 | 3.9 | 167.08 | 15.66 | 9.4×10^-5^ | + |
| 2-Hydroxycinnamic acid | (M+H)^+^ | 165.05393 | 164.04734 | 4.0 | 0.7701 | 1.1 | 298.38 | 1.29 | 1.0×10^-4^ | + |
| Tyramine | (M+H-H_2_O)^+^ | 120.08043 | 137.08406 | -2.6 | 0.9199 | 3.9 | 255.16 | 1.22 | 0.01 | + |
| Urea | (M+H)^+^ | 61.03907 | 60.03236 | 11.2 | 0.9996 | 1.7 | 104.29 | 1.37 | 0.04 | + |
| DG(18:0/20:4) | (M+H-H_2_O)^+^ | 627.53317 | 644.53797 | -0.7 | 0.8605 | 1.6 | 198.13 | 0.79 | 0.03 | + |
| 2-Methylbutyroylcarnitine | (M+H)^+^ | 246.16932 | 245.16271 | 2.71 | 0.9952 | 1.8 | 240.14 | 1.23 | 0.02 | + |
| Hydroxykynurenine | (M+CH_3_COO+2H)^+^ | 285.10842 | 224.07971 | 3.4 | 0.7997 | 1.3 | 383.19 | 1.48 | 0.03 | + |
| 4-Hydroxycinnamic acid | (M+H-H_2_O)^+^ | 147.04313 | 164.04734 | -2.6 | 0.7232 | 1.5 | 298.58 | 1.27 | 1.0×10^-4^ | + |
| *γ*-glutamylalanine | (M+H)^+^ | 219.09704 | 218.09027 | 3.1 | 0.9867 | 1.1 | 407.41 | 1.36 | 0.008 | + |
| Cryptoxanthin | M^+^ | 552.42933 | 552.43312 | -0.7 | 0.9183 | 3.0 | 34.03 | 0.82 | 0.03 | + |
| Cellobiose | (M+NH_4_)^+^ | 360.14905 | 342.11621 | 9.6 | 0.9666 | 1.6 | 392.19 | 1.80 | 0.003 | + |
| Cysteine-S-sulfate | (M+H)^+^ | 201.98313 | 200.97656 | 3.2 | 0.9280 | 1.6 | 314.31 | 2.09 | 1.0×10^-4^ | + |
| Sphingosine 1-phosphate | (M+H)^+^ | 380.25507 | 379.24876 | 1.6 | 0.9545 | 1.5 | 279.89 | 0.80 | 0.01 | + |
| 2,4-Diaminobutyric acid | (M+H-H_2_O)^+^ | 101.07013 | 118.07423 | -3.5 | 0.8366 | 1.7 | 370.47 | 1.25 | 0.02 | + |
| Aminoadipic acid | (M+H)^+^ | 162.07525 | 161.06881 | 4.0 | 0.8100 | 1.2 | 408.00 | 1.31 | 0.04 | + |
| 3-Phenyllactic acid | (M+H-2H_2_O)^+^ | 131.04818 | 166.06299 | -8.9 | 0.8576 | 1.5 | 255.25 | 1.21 | 0.004 | + |
| Lactose | (M+H)^+^ | 343.12286 | 342.11621 | 1.9 | 0.7935 | 1.5 | 399.76 | 1.42 | 0.04 | + |
| L-Methionine sulfoxide | (M+H)^+^ | 166.05226 | 165.04596 | 3.8 | 0.9514 | 1.5 | 363.96 | 1.46 | 0.01 | + |
| Dopamine | (M+H-H_2_O)^+^ | 136.07493 | 153.07897 | -2.6 | 0.9024 | 1.9 | 298.40 | 1.28 | 2.0×10^-4^ | + |
| Ergothioneine | (M+H)^+^ | 230.09541 | 229.08849 | 2.3 | 0.9738 | 1.9 | 323.78 | 1.33 | 0.04 | + |
| Glutamylglutamic acid | (M+H)^+^ | 277.10202 | 276.09575 | 2.3 | 0.9584 | 1.3 | 459.93 | 1.43 | 0.01 | + |
| Glutamylvaline | (M+H)^+^ | 247.12852 | 246.12157 | 2.8 | 0.7770 | 1.3 | 376.01 | 1.31 | 0.007 | + |
| Histamine | (2M+Na)^+^ | 245.1511 | 111.07965 | -3.7 | 0.7175 | 1.5 | 220.13 | 1.64 | 0.02 | + |
| Indole | (M+H)^+^ | 118.06426 | 117.05785 | 5.5 | 0.9220 | 1.8 | 255.84 | 1.23 | 0.02 | + |
| Asparagine | (M+H)^+^ | 133.05979 | 132.05349 | 4.8 | 0.9924 | 1.7 | 374.47 | 1.24 | 0.009 | + |
| Carnitine | (M+H)^+^ | 163.11199 | 162.11302 | -6.9 | 0.9382 | 1.8 | 357.99 | 1.25 | 0.03 | + |
| Kynurenine | (M+H)^+^ | 209.09131 | 208.08478 | 3.1 | 0.9409 | 1.5 | 259.04 | 1.22 | 0.03 | + |
| Norleucine | (2M+H)^+^ | 264.18617 | 131.09463 | -1.2 | 0.9372 | 1.3 | 262.63 | 1.35 | 0.04 | + |
| Palmitoylcarnitine | (M+H)^+^ | 401.34122 | 400.34214 | -0.2 | 0.9903 | 1.3 | 154.82 | 1.30 | 0.02 | + |
| Saccharopine | (M+H)^+^ | 277.13898 | 276.13213 | 2.5 | 0.9410 | 1.3 | 449.84 | 1.51 | 0.03 | + |
| Serine | (M+H)^+^ | 106.04897 | 105.04259 | 6.1 | 0.9631 | 1.5 | 374.35 | 1.24 | 0.004 | + |
| Tyrosine | (M+H)^+^ | 182.08044 | 181.07389 | 3.6 | 0.9658 | 1.1 | 298.28 | 1.26 | 9.0×10^-4^ | + |
| Maltotriose | (M+NH_4_)^+^ | 522.2013 | 504.16903 | 6.4 | 0.9873 | 1.1 | 451.35 | 1.39 | 0.04 | + |
| *N*-Acetylglucosamine | (M+H-2H_2_O)^+^ | 186.07529 | 221.08994 | -6.6 | 0.7332 | 1.6 | 258.59 | 1.56 | 0.04 | + |
| *N-*Acetyllactosamine | (M+H-H_2_O)^+^ | 366.13804 | 383.14276 | -1.2 | 0.9141 | 1.5 | 233.26 | 2.99 | 0.003 | + |
| *N*-Acetylglutamine | (M+CH_3_COO+2H)^+^ | 249.10764 | 188.07971 | 4.2 | 0.7013 | 1.7 | 416.79 | 2.05 | 4.5×10^-6^ | + |
| *N-*Acetylmannosamine | (M+H-H_2_O)^+^ | 204.08619 | 221.08994 | -1.7 | 0.7373 | 1.6 | 259.31 | 1.25 | 8.4×10^-5^ | + |
| *N*-Acetylputrescine | (M+H)^+^ | 131.11692 | 130.11061 | 4.8 | 0.9907 | 1.5 | 322.77 | 1.34 | 0.005 | + |
| N-(o)-Hydroxyarginine | (M+CH_3_CN+H)^+^ | 232.13965 | 190.10659 | 3.4 | 0.8433 | 1.3 | 436.34 | 0.76 | 0.03 | + |
| CerP(d18:1/16:0) | (M+H)^+^ | 618.51829 | 617.47843 | 6.5 | 0.9024 | 1.3 | 34.73 | 0.78 | 0.01 | + |
| Ornithine | (M+H)^+^ | 133.09627 | 132.08988 | 4.8 | 0.9999 | 1.2 | 517.02 | 1.41 | 0.04 | + |
| Pantothenate | (M+H)^+^ | 220.11722 | 219.11067 | 2.9 | 0.7156 | 1.3 | 278.07 | 1.28 | 0.04 | + |
| Glycerylphosphorylethanolamine | (M+H)^+^ | 216.06228 | 215.05587 | 2.9 | 0.9881 | 1.1 | 390.42 | 1.35 | 0.005 | + |
| Stearamide | (M+CH_3_COO+2H)^+^ | 344.31469 | 283.28751 | 2.5 | 0.7178 | 1.2 | 40.27 | 0.62 | 0.001 | + |
| Stearoylcarnitine | M^+^ | 428.37095 | 428.37343 | -0.6 | 0.9940 | 1.8 | 170.26 | 1.26 | 0.008 | + |
| Uracil | (M+H)^+^ | 113.03364 | 112.02728 | 5.7 | 0.7368 | 1.5 | 163.56 | 1.32 | 0.04 | + |
| Uridine | (M+H)^+^ | 245.07613 | 244.06954 | 2.7 | 0.9960 | 1.4 | 163.53 | 1.38 | 0.04 | + |
| *N,N*-dimethylarginine | (M+H)^+^ | 203.1497 | 202.14298 | 3.4 | 0.9831 | 2.2 | 506.43 | 1.65 | 0.01 | + |
| *N6,N6,N6*-Trimethyl-lysine | (M+H)^+^ | 189.15846 | 188.15248 | 3.2 | 0.9959 | 1.8 | 528.31 | 1.71 | 0.01 | + |
| *N*-Acetylneuraminic acid | (M+H)^+^ | 310.11247 | 309.10598 | 2.1 | 0.7864 | 1.34 | 372.21 | 1.29 | 0.02 | + |
| Lysine | (M+CH_3_CN+H)^+^ | 188.13864 | 146.10553 | 4.4 | 0.9802 | 1.1 | 579.97 | 1.41 | 0.003 | + |
| 2-Methyl-3-hydroxybutyric acid | (M-H)^-^ | 117.05514 | 118.06299 | -6.6 | 0.7226 | 1.9 | 162.72 | 1.41 | 0.002 | – |
| 3-Hydroxycapric acid | (M-H)^-^ | 187.1333 | 188.14124 | -4.2 | 0.9868 | 2.5 | 108.33 | 1.29 | 0.02 | – |
| 3-Methoxy-4-hydroxyphenylglycol sulfate | (M-H)^-^ | 263.02202 | 264.03037 | -3.2 | 0.8685 | 1.3 | 46.79 | 1.41 | 0.004 | – |
| alpha-Tocopherol | (M-H)^-^ | 429.37174 | 430.38108 | -2.2 | 0.9490 | 1.3 | 33.10 | 0.78 | 4.0×10^-4^ | – |
| Chenodeoxycholate | (M-H)^-^ | 391.28391 | 392.29266 | -2.5 | 0.9999 | 2.5 | 163.86 | 1.96 | 0.002 | – |
| Cholesterol sulfate | (M-H)^-^ | 465.30383 | 466.31168 | -1.7 | 1.0000 | 10.1 | 26.84 | 1.36 | 0.002 | – |
| Glycochenodeoxycholate | (M-H)^-^ | 448.30601 | 449.31412 | -1.8 | 1.0000 | 12.1 | 213.89 | 12.84 | 4.9×10^-6^ | – |
| Glycocholic acid | (M-H)^-^ | 464.30102 | 465.30904 | -1.7 | 1.0000 | 4.8 | 256.29 | 12.22 | 2.0×10^-4^ | – |
| Palmitoleic acid | (M-H)^-^ | 253.21679 | 254.22458 | -3.1 | 0.9999 | 10.3 | 51.28 | 1.31 | 0.002 | – |
| Enterostatin | (M-H)^-^ | 495.27673 | 496.27578 | 1.9 | 0.8730 | 1.6 | 30.02 | 1.57 | 7.5×10^-6^ | – |
| Homocysteic acid | (M+Na-2H)^-^ | 203.99648 | 183.02014 | -12.9 | 0.9679 | 1.7 | 46.13 | 0.64 | 4.0×10^-4^ | – |
| Maslinic Acid | (M-H)^-^ | 471.34581 | 472.35526 | -2.0 | 0.9679 | 1.3 | 105.33 | 1.34 | 0.002 | – |
| 9,10-Epoxyoctadecanoic acid | (M-H)^-^ | 313.24237 | 314.24571 | -1.1 | 0.9765 | 2.8 | 60.55 | 1.29 | 0.006 | – |
| Estrone sulfate | (M-H)^-^ | 349.1104 | 350.11879 | -2.4 | 0.9976 | 1.4 | 28.21 | 1.70 | 0.02 | – |
| Oleic acid | (M-H)^-^ | 281.24799 | 282.25588 | -2.8 | 1.0000 | 2.9 | 106.57 | 1.16 | 0.02 | – |
| Pregnenolone sulfate | (M-H)^-^ | 395.18808 | 396.19704 | -2.3 | 0.9497 | 1.1 | 63.34 | 1.97 | 0.008 | – |
| Succinate | (M-H)^-^ | 117.01897 | 118.02661 | -6.5 | 0.9794 | 1.5 | 394.92 | 1.41 | 7.5×10^-5^ | – |
| Taurochenodeoxycholate | (M-H)^-^ | 498.28861 | 499.29675 | -1.6 | 1.0000 | 14.0 | 163.31 | 18.39 | 7.1×10^-6^ | – |
| Xanthine | (M-H)^-^ | 151.02567 | 152.03343 | -5.1 | 0.8809 | 1.9 | 224.30 | 1.81 | 1.0×10^-4^ | – |
| Ketoisocaproic acid | (M-H)^-^ | 129.05512 | 130.06299 | -6.1 | 0.9247 | 3.6 | 108.99 | 0.67 | 0.02 | – |
| Gulonic acid | (M-H_2_O-H)^-^ | 177.03993 | 196.05830 | -9.4 | 0.8477 | 2.2 | 133.29 | 0.82 | 0.02 | – |
| Valeric acid | (M-H)^-^ | 101.06044 | 102.06808 | -7.5 | 0.8597 | 1.5 | 67.09 | 0.81 | 0.003 | – |
| PS (18:0/18:1) | (M-H)^-^ | 788.52625 | 789.55198 | -3.3 | 0.8939 | 1.2 | 144.13 | 0.82 | 0.03 | – |
| 2-Oxoadipic acid | (M-H_2_O-H)^-^ | 141.01712 | 160.03717 | -12.5 | 0.9682 | 14.1 | 345.95 | 0.81 | 0.001 | – |
| 7-Methylxanthine | (M-H)^-^ | 165.04063 | 166.04908 | -5.1 | 0.7202 | 1.5 | 120.59 | 0.73 | 0.02 | – |
| Acetylcarnitine | (M-H)^-^ | 203.10767 | 204.12358 | -7.8 | 0.9326 | 1.3 | 190.74 | 1.56 | 0.008 | – |
| Glucose | (M-H)^-^ | 179.05565 | 180.06338 | -4.3 | 0.9787 | 1.7 | 397.08 | 1.21 | 0.007 | – |
| Biliverdin | (M-H)^-^ | 581.23943 | 582.24783 | -1.4 | 0.9832 | 1.2 | 242.37 | 0.82 | 0.03 | – |
| Galacturonic acid | (M-H)^-^ | 193.03481 | 194.04265 | -4.0 | 0.9297 | 1.1 | 392.84 | 1.78 | 5.7×10^-6^ | – |
| Eicosapentaenoic acid | (M+CH_3_COO)^-^ | 361.23658 | 302.22458 | -2.6 | 0.7076 | 1.1 | 108.03 | 1.24 | 2.0×10^-4^ | + |
| Estrone glucuronide | (M-H)^-^ | 445.18538 | 446.19407 | -1.9 | 0.8910 | 1.1 | 207.94 | 1.44 | 0.02 | – |
| Formylanthranilic acid | (M-H)^-^ | 164.03476 | 165.04259 | -4.7 | 0.8244 | 1.2 | 103.20 | 0.74 | 0.005 | – |
| Galactonic acid | (M+CH3COO)^-^ | 255.07124 | 196.05830 | -3.6 | 0.9094 | 1.1 | 303.39 | 0.81 | 0.04 | – |
| Glycolithocholic acid | (M-H)^-^ | 432.3099 | 433.31920 | -0.9 | 0.9994 | 1.8 | 176.51 | 4.69 | 0.002 | – |
| Guanosine | (M-H)^-^ | 282.08344 | 283.09167 | -2.9 | 0.9779 | 1.3 | 265.85 | 0.76 | 0.02 | – |
| Hydroxyphenyllactic acid | (M-H)^-^ | 181.04992 | 182.05791 | -4.4 | 0.7792 | 1.3 | 202.23 | 1.21 | 0.04 | – |
| Indoxyl sulfate | (M-H)^-^ | 212.00214 | 213.00958 | -3.5 | 0.9911 | 11.6 | 29.81 | 0.79 | 0.02 | – |
| Inosine | (M-H)^-^ | 267.07367 | 268.08077 | -2.6 | 0.9939 | 1.2 | 312.86 | 1.43 | 0.01 | – |
| Ascorbic acid | (M+CH_3_COO)^-^ | 235.04487 | 176.03208 | -4.1 | 0.9019 | 1.3 | 133.26 | 0.81 | 0.03 | + |
| Aspartate | (M-H)^-^ | 132.02979 | 133.03751 | -5.8 | 0.9409 | 1.3 | 326.62 | 1.88 | 4.9×10^-5^ | – |
| Histidine | (M-H)^-^ | 154.06172 | 155.06948 | -5.0 | 0.8932 | 1.9 | 401.64 | 1.22 | 0.008 | – |
| Lithocholic acid | (M-H)^-^ | 375.28854 | 376.29775 | -2.4 | 0.9735 | 1.2 | 89.43 | 1.65 | 0.02 | – |
| Pipecolic acid | (M-H)^-^ | 128.07112 | 129.07898 | -6.1 | 0.9997 | 1.6 | 274.96 | 0.73 | 0.001 | – |
| Threonic acid | (M-H)^-^ | 135.02944 | 136.03717 | -5.7 | 0.9688 | 1.4 | 332.32 | 1.65 | 9.7×10^-5^ | – |
| Threonine | (M-H)^-^ | 118.05032 | 119.05824 | -6.7 | 0.9026 | 1.1 | 361.43 | 1.30 | 0.001 | – |
| myo-Inositol | (M-H_2_O-H)^-^ | 161.04466 | 180.06339 | -10.4 | 0.8236 | 1.5 | 126.63 | 1.39 | 0.004 | – |
| *N*-Acetylaspartic acid | (M-H)^-^ | 174.04032 | 175.04807 | -4.4 | 0.8952 | 1.2 | 399.66 | 1.23 | 0.03 | – |
| *N*-Formyl-L-methionine | (M-H)^-^ | 176.0379 | 177.04596 | -4.6 | 0.8309 | 1.2 | 204.14 | 0.77 | 0.008 | – |
| 5'-Methylthioadenosine | (M-H)^-^ | 296.08132 | 297.08956 | -2.8 | 0.9556 | 1.4 | 104.18 | 2.15 | 0.02 | – |
| Taurine | (M-H)^-^ | 124.0073 | 125.01466 | -5.9 | 0.9975 | 1.1 | 299.22 | 0.83 | 0.03 | – |
| Tetracosanoic acid | (M-H)^-^ | 367.3554 | 368.36543 | -2.7 | 0.9627 | 1.5 | 39.68 | 1.32 | 0.003 | – |
| Tricosanoic acid | (M-H)^-^ | 353.34063 | 354.34978 | -2.6 | 0.9984 | 1.3 | 39.65 | 1.21 | 0.02 | – |
| Uric acid | (M-H)^-^ | 167.02031 | 168.02834 | -4.8 | 0.9337 | 1.1 | 332.40 | 1.60 | 1.9×10^-5^ | – |
| Estriol-16-glucuronide | (M-H)^-^ | 463.19557 | 464.20463 | -1.9 | 0.9986 | 1.1 | 215.09 | 1.31 | 0.03 | – |
| Bilirubin | (M-H)^-^ | 583.25501 | 584.26348 | -1.4 | 0.9669 | 1.2 | 67.76 | 1.53 | 0.002 | – |
| Glutamine | (M-H)^-^ | 145.0619 | 146.0691 | -4.9 | 0.8927 | 2.2 | 378.98 | 1.21 | 0.009 | – |
| Proline | (M-H)^-^ | 114.0559 | 115.06332 | -6.4 | 0.9976 | 1.2 | 313.98 | 1.23 | 0.01 | – |

^*^ VIP, variable importance in projection; ^†^ RT, retention time; ^‡^ FC, fold change (ICP/normal group); ^§^ ESI, electrospray ionization
